# An explainable supervised machine learning predictor of acute kidney injury after adult deceased donor liver transplantation

**DOI:** 10.1186/s12967-021-02990-4

**Published:** 2021-07-28

**Authors:** Yihan Zhang, Dong Yang, Zifeng Liu, Chaojin Chen, Mian Ge, Xiang Li, Tongsen Luo, Zhengdong Wu, Chenguang Shi, Bohan Wang, Xiaoshuai Huang, Xiaodong Zhang, Shaoli Zhou, Ziqing Hei

**Affiliations:** 1 Department of Anesthesiology, The Third Affiliated Hospital of Sun Yat-Sen University, No. 600 Tianhe Road, Guangzhou, Guangdong China; 2 Guangzhou AID Cloud Technology Co., LTD, Guangzhou, Guangdong China; 3 Department of Clinical Data Center, The Third Affiliated Hospital of Sun Yat-Sen University, Guangzhou, Guangdong China; 4 Department of Information, The Third Affiliated Hospital of Sun Yat-Sen University, Guangzhou, Guangdong China; 5 Department of Anesthesiology, The Third Affiliated Hospital of Sun Yat-Sen University, Yuedong Hospital, Meizhou, Guangdong China

**Keywords:** Kidney dysfunction, Liver transplant, SHapley Additive exPlaination methods, SHAP value, Gradient boosting machine, Perioperative medicine, Big data, Artificial intelligence, Prognostic predictor, Clinical assisting tool

## Abstract

**Background:**

Early prediction of acute kidney injury (AKI) after liver transplantation (LT) facilitates timely recognition and intervention. We aimed to build a risk predictor of post-LT AKI via supervised machine learning and visualize the mechanism driving within to assist clinical decision-making.

**Methods:**

Data of 894 cases that underwent liver transplantation from January 2015 to September 2019 were collected, covering demographics, donor characteristics, etiology, peri-operative laboratory results, co-morbidities and medications. The primary outcome was new-onset AKI after LT according to Kidney Disease Improving Global Outcomes guidelines. Predicting performance of five classifiers including logistic regression, support vector machine, random forest, gradient boosting machine (GBM) and adaptive boosting were respectively evaluated by the area under the receiver-operating characteristic curve (AUC), accuracy, F1-score, sensitivity and specificity. Model with the best performance was validated in an independent dataset involving 195 adult LT cases from October 2019 to March 2021. SHapley Additive exPlanations (SHAP) method was applied to evaluate feature importance and explain the predictions made by ML algorithms.

**Results:**

430 AKI cases (55.1%) were diagnosed out of 780 included cases. The GBM model achieved the highest AUC (0.76, CI 0.70 to 0.82), F1-score (0.73, CI 0.66 to 0.79) and sensitivity (0.74, CI 0.66 to 0.8) in the internal validation set, and a comparable AUC (0.75, CI 0.67 to 0.81) in the external validation set. High preoperative indirect bilirubin, low intraoperative urine output, long anesthesia time, low preoperative platelets, and graft steatosis graded NASH CRN 1 and above were revealed by SHAP method the top 5 important variables contributing to the diagnosis of post-LT AKI made by GBM model.

**Conclusions:**

Our GBM-based predictor of post-LT AKI provides a highly interoperable tool across institutions to assist decision-making after LT.

**Graphic abstract:**

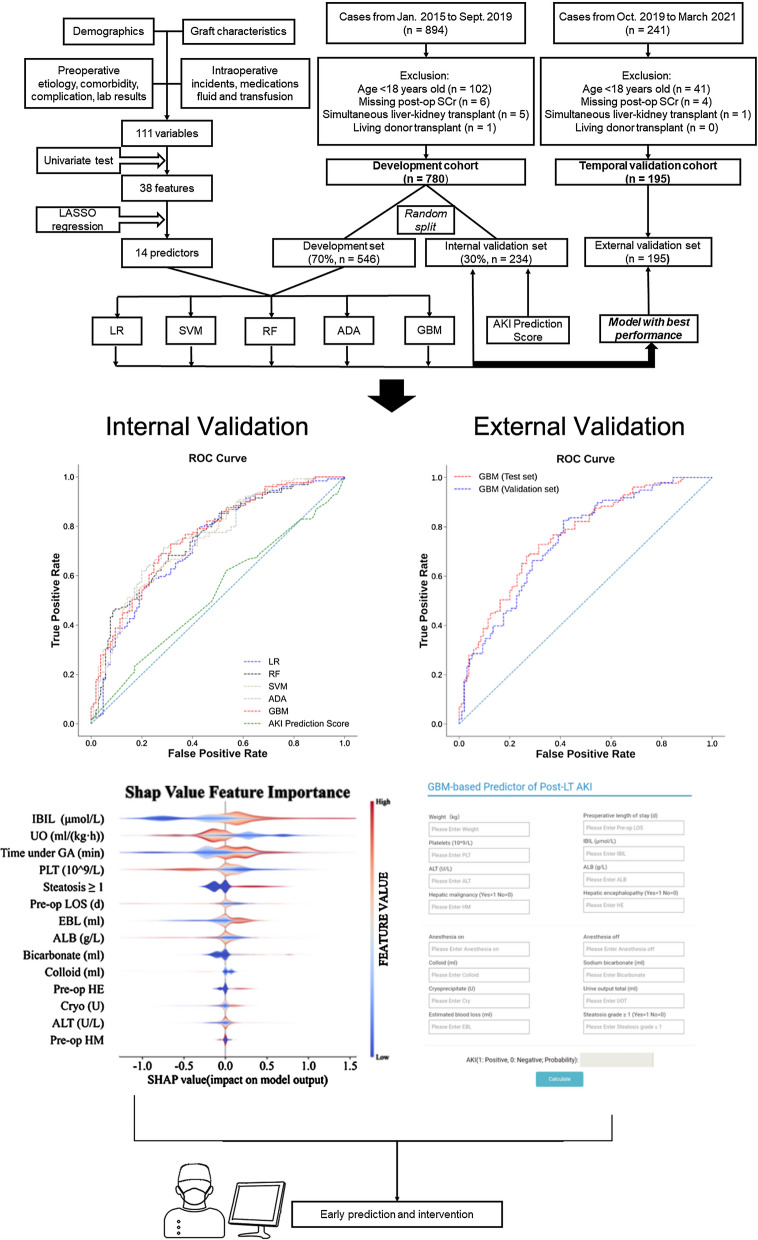

**Supplementary Information:**

The online version contains supplementary material available at 10.1186/s12967-021-02990-4.

## Introduction

Acute kidney injury (AKI) after liver transplantation (LT) holds unique etiology and risk factors compared to AKI in other clinical settings. The reported incidence of post-LT AKI, which derived from various diagnostic criteria, varies from 17 to 95% [1, 2], with an average around 40.7% [3]. Kollmann et al. demonstrated that when using KDIGO criteria, the incidence of post-LT AKI observed was 61% in the DCD group and 40% in the DBD group [2]*.* AKI after LT is associated with increased post-operative mortality, potential progression to chronic kidney disease (CKD), longer length of stay and increased medical expenditure [1].Graft characteristics, intraoperative hemodynamic instability and post-operative exposure to nephrotoxic immunosuppression have been considered to be associated with AKI after LT [4–6]. Early interventions like perioperative continuous renal replacement therapy (CRRT) and restraint on nephrotoxic medications shall be considered in patients with AKI, but the timing of such decisions depends largely on personal experience and a reliable predicting model can greatly facilitate these decisions [7].

Machine learning (ML) algorithms have demonstrated satisfactory performance in building robust predictive models of inpatient AKI [8]. However, many of these studies fed relatively abundant features to ML algorithms without dimensionality reduction [9]. Highly correlated features without regularization are of limited utility in enhancing the predictive power of the model [10]. Moreover, high dimensional features are susceptible to missing data once being externally validated across institutions, hindering clinical application of these models. With current surge of these ML-derived clinical assisting tool [11, 12], criteria for evaluation and regulation of such predictive algorithms have been advocated, which include setting meaningful endpoints and appropriate benchmarks, and ensuring generalizability among institutions [13].

Besides these criteria, relational validity of ML-derived predictive models, that is, the extent to which physicians can interpret them, has been emphasized lately, since a sound statistical validity does not necessarily guarantee the usability of these models [14]. The “black magic” of ML remains to be debated for the difficulty to understand the mechanisms driving within [15]. SHapley Additive exPlanations (SHAP) method developed by Lundberg [16] is a Game Theory-based method, within which the individual features act as players in a prediction task and the Shapley value helps to fairly distribute the prediction performance among the features [17]. This method enables black-box ML algorithms to be explained on individual level. In this study we aimed to select a ML classifier that outperforms statistically in predicting post-LT AKI and further visualize the decision made by ML algorithms to clinicians to assist their decisions. Meanwhile we also validated an AKI prediction score developed by Kalisvaart et al. [5] with our data set and compared the performance of our ML model to this score.

## Experimental procedures

### Source of data and participants

This was a retrospective, single center research conducted in The Third Affiliated Hospital of Sun Yat-sen University-Lingnan Hospital. This study was approved by the Ethnic Committee of the Third Affiliated Hospital of Sun Yat-sen University (NO. [2019]02-609-01), with waiver of informed consent.

Medical data collected by natural language process module from EMRs included demographic data, daily documentation, laboratory and imaging results, anesthesia records, medications, interventions and diagnosis [18]. Donor characteristics were manually collected from the China Organ Transplant Response Systems (CORS, www.cot.org.cn). All data were anonymized. This study is reported as per the Transparent Reporting of a Multivariable Prediction Model for Individual Prognosis or Diagnosis (TRIPOD) guidelines [19].

As a result, data of 894 cases that underwent LT from January 2015 to September 2019 were extracted. After excluding pediatric cases, simultaneous liver-kidney transplantation, living donor transplantation and cases that lack sufficient post-operative records of serum creatinine (SCr), 780 cases were included in the primary cohort for model development and internal validation. Since recipients with impaired pre-transplant renal function are prioritized during organ allocation determined by the model of end-stage liver disease (MELD) score [5], and around 90% of these patients can recover after transplantation [20], we agreed with including patients with preoperative renal injury or diagnosed with hepato-renal syndrome, out of the purpose to predict new onset AKI simply associated with perioperative treatment. As for survival analysis, the end of follow-up was set at December 31st, 2019. Data of patients that underwent deceased donor liver transplantation meeting the same inclusion criteria during October 2019 to March 2021 were exclusively extracted for external validation.

### Perioperative treatment

The grafts were procured from either donation after circulatory death (DCD), donation after brain death (DBD) or donation after brain death followed by circulatory death (DBCD) [21]. *No organs from executed prisoners were used*. The implantation technique consisted of piggyback, standard and split liver transplantation. Liver biopsy samples were collected before and after graft reperfusion. Intraoperative extracorporeal venovenous bypass was hardly applied since it was not significantly advantageous [22]. Transfusion, fluid management and use of vasoactive and hemostatic agent were adjusted according to an overall assessment of volume balance and hemodynamic stability. Boluses of vasoactive agents were mostly given to counter post-reperfusion syndrome, otherwise continuous infusion were preferred. Colloids were only used during reperfusion phase when coagulation deficiency was corrected and satisfactory urine output was observed. For patients receiving ABO-incompatible graft, Tacrolimus introduction was initiated at Day 2 after the surgery, otherwise a renal sparing therapy that initiated Tacrolimus at Day 4 was adopted. A detailed description of anesthesia and immunotherapy can be found in Additional file 4: Appendix S4.

### Outcome

The primary outcome was postoperative AKI, diagnosed within 7 days post-operatively according to the criteria proposed by The Kidney Disease: Improving Global Outcomes (KDIGO) guideline [23] (Additional file 5). Criteria concerning urine output in KDIGO guideline were not adopted, since it required urine output to be less than 0.5 ml·kg^−1^·h^−1^ for 6 h to diagnose AKI, which was not as timely as the SCr result obtained immediately after the surgery. Moreover, for patients receiving LT we tested post-operative SCr on a daily basis, which was sufficient to identify AKI within one week after the surgery.

### Predictors and selection

A total of 111 variables were chosen for initial analysis (Additional file 1: Appendix S1, Table S2), mainly covering demographics and donor characteristics; preoperative comorbidities, laboratory values, etiology of liver and complications; intraoperative incidents, medication, fluid infusion and blood product transfusion; post-operative medications. Certain categorical variables were generated by imposing specific rules according to their definitions (Additional file 1: Appendix S1, Table S1). MELD score was calculated according to the standard of the United Network for Organ Sharing (UNOS) Liver and Intestinal Organ Transplantation Committee (Additional 6). Graft steatosis was graded according to Nonalcoholic Steatohepatitis Clinical Research Network (NASH CRN) (https://jhuccs1.us/nash/).

For variables with a missing proportion less than 10%, we imputed categorical variables with the mode and continuous variable with Multivariate Imputation by Chained Equations (MICE) algorithm [24]. To minimize potential over-fitting brought by high dimensionality of the features, only features that were statistically significant (*p* < 0.05) in univariate test were chosen and subjected to a least absolute shrinkage and selection operator (LASSO) regression approach. Finally, features with non-zero coefficients after LASSO regression were used to build our models (Additional file 3: Appendix S3, Table S4).

### Statistics

Data cleaning was conducted using Python (Anaconda Distribution, Version 3.7) package. Pandas and Numpy. Scikit-learn (https://github.com/scikit-learn/scikit-learn) package was used to build base models including logistic regression (LR), support vector machine (SVM), random forest (RF), gradient boosting machine (GBM) implemented by decision tree and adaptive boosting (ADA). We also calculated Kalisvaart’s AKI prediction score that use donor and recipient body mass index (BMI), DCD grafts, plasma requirements, and recipient warm ischemic time (WIT) as variables for risk stratification [5].

The primary cohort was randomly separated into 70% development set and 30% internal validation set. Bootstrap method was implemented 1000 times on internal validation set to derive confidence interval of AUC, accuracy, sensitivity and specificity. Grid search method with five-fold cross validation was used to choose best hyperparameters for each model (Additional file 2: Appendix S2, Table S1). Mean with standard deviation, or median with interquartile range was used to analyze and express continuous variables, the comparisons of which were made using the Independent-sample T test or Mann–Whitney U test. Categorical variables were expressed in quantities and percentages and compared by the Chi-square test. Post-operative survival was estimated by Kaplan–Meier methods and examined by Gehan-Breslow-Wilcoxon test. SHAP method was implemented using Python shap package (https://shap.readthedocs.io/en/latest/).

## Results

### Baseline characteristics of the participants

The internal validation set consisted of a majority of male (n = 682, 87.44%), with a mean age of 50.7 years and BMI around 22.78 (Table 1). Among the 780 cases included, 430 (55.13%) were diagnosed with AKI (AKI group), within which 159 cases (36.97%) were stage 3 AKI requiring postoperative CRRT.

**Table 1:** Characteristics, diagnosis and perioperative features of current cohort

	All (N = 780)	Non-AKI (n = 350)	AKI(n = 430)	P value
Age (y)	50.719 (10.638)	51.051 (10.433)	50.449 (10.808)	0.295
Height (cm)	167.954 (9.065)	167.734 (6.428)	168.134 (10.753)	0.052
Weight (kg)	64.628 (11.304)	63.404 (10.889)	65.628 (11.548)	0.004
Body Mass Index	22.782 (3.574)	22.539 (3.529)	22.98 (3.602)	0.018
Preoperative LOS (d)	11 (2–26)	14 (4–28)	8 (2–23)	0.001
Diagnosis of AKI				
No AKI		350.0 (100.0%)	/	
Stage 1 AKI		/	177.0 (41.163%)	
Stage 2 AKI		/	63.0 (14.651%)	
Stage 3 AKI		/	190.0 (44.186%)	
Stage 3 AKI requring CRRT		/	159.0 (36.977%)	
AKI diagnosis during POD1		/	288 (66.977%)	
Preoperative renal function				
CKD (n)	121.0 (15.513%)	49.0 (14.0%)	72.0 (16.744%)	0.34
AKI (n)	172.0 (22.051%)	67.0 (19.143%)	105.0 (24.419%)	0.093
HRS (n)	33.0 (4.231%)	8.0 (2.286%)	25.0 (5.814%)	0.024
SCr (μmol/L)	91.777 (70.334)	92.388 (68.852)	91.28 (71.593)	0.047
BUN (mmol/L)	6.846 (5.823)	6.56 (5.218)	7.078 (6.268)	0.985
eGFR (ml/(min*1.73^2^))	95.029 (32.145)	93.749 (29.966)	96.07 (33.813)	0.127
SCr_Mean (μmol/L)	79.343 (71.641)	75.837 (65.256)	82.197 (76.402)	0.917
Use of CRRT (n)	94.0 (12.051%)	24.0 (6.857%)	70.0 (16.279%)	< 0.001
Frequency of CRRT (times)	2.567 (10.727)	1.351 (8.312)	3.556 (12.269)	< 0.001
Preoperative laboratory values				
HCT	0.299 (0.076)	0.312 (0.08)	0.288 (0.07)	< 0.001
PLT(10^9^/L)	96.026 (79.4)	116.597 (95.149)	79.281 (58.79)	< 0.001
ALT (U/L)	126.282 (399.834)	90.349 (235.856)	155.53 (493.081)	0.004
AST (U/L)	172.242 (538.996)	148.429 (369.227)	191.626 (644.817)	< 0.001
TBIL (μmol/L)	250.278 (249.713)	172.311 (217.596)	313.739 (256.351)	< 0.001
DBIL (μmol/L)	159.74 (168.516)	116.107 (152.227)	195.256 (172.907)	< 0.001
IBIL (μmol/L)	90.537 (96.523)	56.204 (72.764)	118.483 (104.24)	< 0.001
ALB (g/L)	35.668 (4.906)	36.212 (5.283)	35.225 (4.535)	0.023
PT (s)	25.16 (13.483)	21.115 (9.851)	28.452 (15.064)	< 0.001
APTT (s)	54.653 (20.923)	49.183 (16.041)	59.105 (23.267)	< 0.001
FIB (g/L)	1.982 (1.422)	2.357 (1.372)	1.676 (1.39)	< 0.001
INR	2.339 (1.574)	1.912 (1.397)	2.686 (1.625)	< 0.001
Etiology of liver				
Hepatitis B (n)	577.0 (73.974%)	257.0 (73.429%)	320.0 (74.419%)	0.817
Hepatitis C (n)	17.0 (2.179%)	11.0 (3.143%)	6.0 (1.395%)	0.157
Dual infection (n)	9.0 (1.154%)	5.0 (1.429%)	4.0 (0.93%)	0.756
Hepatic Malignancy (n)	312.0 (40.0%)	190.0 (54.286%)	122.0 (28.372%)	< 0.001
Cirrhosis (n)	623.0 (79.872%)	292.0 (83.429%)	331.0 (76.977%)	0.032
Preoperative complications				
MELD score	24 (22–35)	22(22–29)	30 (22–38)	< 0.001
Portal hypertension (n)	407.0 (52.179%)	192.0 (54.857%)	215.0 (50.0%)	0.201
Ascites (n)	321.0 (41.154%)	142.0 (40.571%)	179.0 (41.628%)	0.822
HE (n)	180.0 (23.077%)	41.0 (11.714%)	139.0 (32.326%)	< 0.001
Plasmapheresis (n)	7.0 (0.897%)	2.0 (0.571%)	5.0 (1.163%)	0.625
HPS (n)	4.0 (0.513%)	1.0 (0.286%)	3.0 (0.698%)	0.766
ARDS (n)	7.0 (0.897%)	3.0 (0.857%)	4.0 (0.93%)	0.784
ALI (n)	0.0 (0.0%)	0.0 (0.0%)	0.0 (0.0%)	1
MV (n)	49.0 (6.282%)	9.0 (2.571%)	40.0 (9.302%)	< 0.001
ICU stay (n)	439.0 (56.282%)	164.0 (46.857%)	275.0 (63.953%)	< 0.001
Hypernatremia (n)	44.0 (5.641%)	10.0 (2.857%)	34.0 (7.907%)	0.004
Metabolic acidosis (n)	336.0 (43.077%)	144.0 (41.143%)	192.0 (44.651%)	0.362
Donor characteristics				
Donor age (y)	39.191 (13.966)	38.894 (14.392)	39.433 (13.621)	0.755
Donor BMI	22.578 (3.199)	22.336 (3.185)	22.779 (3.201)	0.074
ABO incompatibility (n)	120.0 (15.385%)	38.0 (10.857%)	82.0 (19.07%)	0.002
Donor Type				0.248
DBD (n)	448 (57.436%)	212 (60.571%)	236 (54.884%)	
DCD (n)	324 (41.538%)	134 (38.286%)	190 (44.186%)	
DBCD (n)	8 (1.026%)	4 (1.143%)	4 (0.93%)	
Steatosis of donor liver				0.002
Steatosis grade 0 (n)	529 (67.821%)	260.0 (74.286%)	269 (62.558%)	
Steatosis grade 1 (n)	170 (21.795%)	62.0 (17.714%)	108 (25.116%)	
Steatosis grade 2 (n)	35 (4.487%)	9.0 (2.571%)	26 (6.047%)	
Steatosis grade 3 (n)	1 (0.128%)	0.0 (0.0%)	1 (0.233%)	
Steatosis grade ≥ 1	206.0 (26.41%)	71.0 (20.286%)	135.0 (31.395%)	0.001
Steatosis grade ≥ 2	36.0 (4.615%)	9.0 (2.571%)	27.0 (6.279%)	0.022
Lack of pathology assesment (n)	45 (5.769%)	19 (5.429%)	26 (6.046%)	0.721
Surgery characteristics				
Time of surgery (min)	442.713 (92.854)	425.297 (87.949)	456.888 (94.418)	< 0.001
Time under anesthesia (min)	538.888 (97.864)	519.56 (92.679)	554.621 (99.251)	< 0.001
Recipient warm ischemic time (min)	46.45 (12.035)	45.919 (12.183)	46.883 (11.909)	0.088
Cold ischemic time (h)	6.255 (1.358)	6.226 (1.393)	6.278 (1.329)	0.476
Surgical technique				0.304
Piggyback (n)	713 (91.41%)	317 (90.571%)	396 (92.093%)	
Split liver (n)	36 (4.615%)	15 (4.286%)	21 (4.884%)	
Standard (n)	31 (3.974%)	18 (5.143%)	13 (3.023%)	
Intraoperative fluid and transfusion				
Crystalloid (ml)	2618.423 (2240.489)	2775.575 (2366.817)	2490.944 (2126.798)	0.094
Colloid (ml)	124.26 (427.879)	153.448 (424.742)	100.583 (429.443)	0.006
Albumin (ml)	218.295 (116.74)	222.629 (111.083)	214.779 (121.15)	0.483
Transfusion				
RBC (ml)	1500.39 (1318.45)	1279.989 (1333.507)	1679.177 (1280.024)	< 0.001
Plasma (ml)	1862.806 (1613.71)	1725.862 (1376.393)	1973.893 (1777.029)	0.063
Cryoprecipitate (U)	30.276 (15.83)	27.359 (14.9)	32.653 (16.182)	< 0.001
EBL (ml)	2051.489 (2027.519)	1679.857 (1890.832)	2354.685 (2086.165)	< 0.001
Urine output (ml·kg^−1^·h^−1^)	3.104 (2.146)	3.708 (2.219)	2.613 (1.954)	< 0.001
Ascites removal (ml)	959.665 (1889.757)	947.011 (1997.938)	969.93 (1799.531)	0.196
Intraoperative medication				
rFVIIa (mg)	0.346 (1.127)	0.211 (1.03)	0.455 (1.19)	< 0.001
Prothrombin complex concentrate (IU)	587.692 (433.693)	554.857 (434.497)	614.419 (431.7)	0.043
Fibrinogen (g)	0.404 (1.293)	0.342 (0.735)	0.453 (1.609)	0.567
Terlipressin (mg)	0.322 (0.551)	0.195 (0.447)	0.426 (0.604)	< 0.001
Norepinephrine, bolus (mg)	0.008 (0.022)	0.006 (0.018)	0.009 (0.024)	0.353
Epinephrine, bolus (mg)	0.028 (0.299)	0.011 (0.161)	0.042 (0.376)	0.785
Dopamine, bolus (mg)	12.0 (1.538%)	4.0 (1.143%)	8.0 (1.86%)	0.874
Bicarbonate (ml)	127.006 (234.266)	89.429 (221.225)	157.593 (240.316)	< 0.001
Use of norepinephrine, continuous (n)	649.0 (83.205%)	301.0 (86.0%)	348.0 (80.93%)	0.074
Use of epinephrine, continuous (n)	553.0 (70.897%)	250.0 (71.429%)	303.0 (70.465%)	0.829
Use of dopamine, continuous (n)	245.0 (31.41%)	106.0 (30.286%)	139.0 (32.326%)	0.594
Use of aramine (n)	34.0 (4.359%)	7.0 (2.0%)	27.0(6.279%)	0.006
Intraoperative incident				
Cardiac arrest (n)	21.0 (2.692%)	3.0 (0.857%)	18.0(4.186%)	0.008
Acidosis (n)	322.0 (41.282%)	133.0 (38.0%)	189.0 (43.953%)	0.108
Hypotension (n)	649.0 (83.205%)	298.0 (85.143%)	351.0 (81.628%)	0.226

BMI, body mass index; LOS, length of stay; MELD, model for end stage liver disease. CRRT, continuous renal replacement therapy; ARDS, acute respiratory distress syndrome;ICU, intensive care unit; HCT, hematocrit; PLT, platelets; WBC, white blood cell; ALT, alanine transaminase; AST, aspartate transaminase; TBIL, total bilirubin; DBIL, direct bilirubin; IBIL, indirect bilirubin; ALB, albumin; SCr, serum creatinine; BUN, blood urea nitrogen; PT, prothrombin time; APTT, activated partial thromboplastin time; FIB, fibrinogen; INR, international normalized ratio; eGFR, estimated glomerular filtration rate; DBD, donation after brain death; DCD, donation after circulatory death; DBCD, donation after brain death followed by circulatory death; GA, general anesthesia; RBC, red blood cell; EBL, estimated blood loss; rFVIIa, recombinant activated factor VII

Patients that did not end up with AKI (Non-AKI group) presented comparable percentage of preoperative AKI and CKD to that of AKI group. With evident use of CRRT in AKI group (16.27% vs. 6.85%, *p* < 0.001), the biomarkers of renal function were not significantly different in clinical settings. Meanwhile, AKI group presented more severe liver dysfunction and coagulopathy, and higher MELD score (median 30 vs. 22, *p* < 0.001). AKI group also held less cases with hepatic malignancy (28.37% vs. 54.28%, *p* < 0.001) and higher the percentage of hepatic encephalopathy (HE) (32.33% vs. 11.71%, *p* < 0.001). The percentage of graft steatosis and ABO incompatibility were also significantly higher in AKI group.

During LT, AKI group tended to suffer from greater blood loss and required higher volume of blood transfusion, higher dose of terlipressin, sodium bicarbonate and hemostatic medications. Consistently, the average intraoperative urine output of AKI group was significantly lower (mean 2.61 vs. 3.70 ml·kg^−1^·h^−1^, *p* < 0.001).

A great majority of AKI cases (n = 288, 66.97%) were diagnosed within 24 h after LT (Table 1), that is, prior to the introduction of Tacrolimus. Although we collected data of post-operative medications prior to the appearance of diagnostic SCr (for AKI group) or prior to the record of maximum SCr (for Non-AKI group) (Additional file 3: Appendix S3, Table S3), the heterogeneity in the timing of diagnosis made them unsuitable as predictors in our model.

**Fig. 1:**
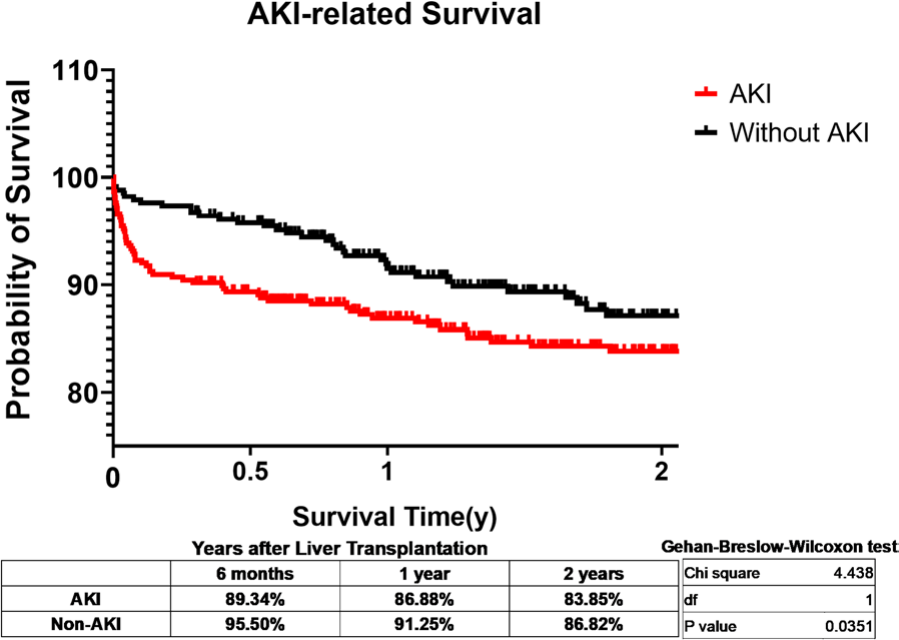
Postoperative survival associated with AKI. Patients with post-LT AKI demonstrated significantly lower survival, especially during the first 6 months after surgery

The 6-month, 1-year and 2-year survival of patients in AKI group were respectively 89.34%, 86.88% and 83.85%, which was significantly lower compared to Non-AKI group (95.50%, 91.25% and 86.82%) (Fig. 1) ( 5: ).

### Internal validation performance

**Fig. 2:**
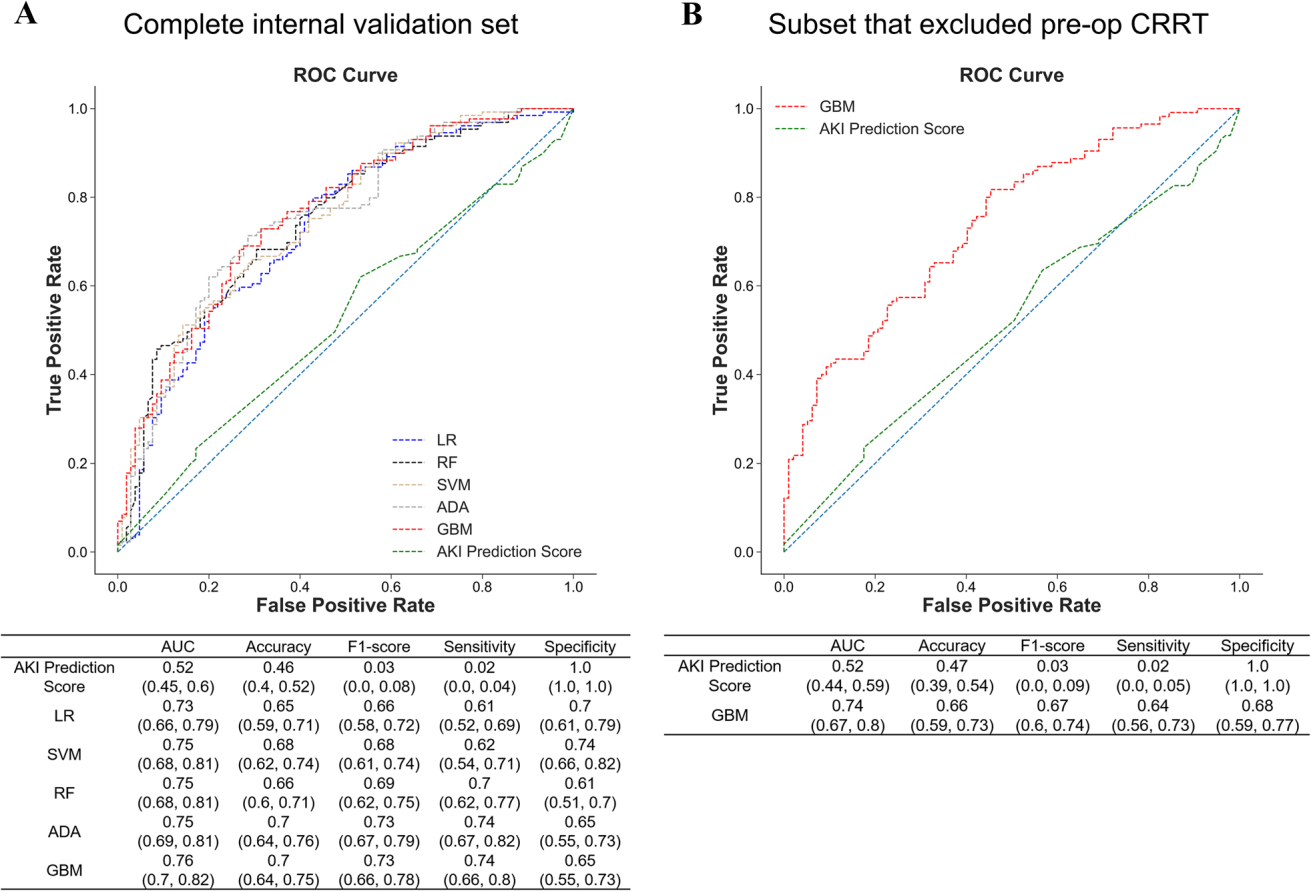
Performance of machine learning models and AKI prediction score.** A** Performance of all predicting models in the internal validation set, which included patients requiring preoperative CRRT. **B** Performance of GBM model and AKI prediction score in a subset that excluded patients requiring preoperative CRRT, to conform to the exclusion criteria in Kalisvaart’s study when they designed this score

Finally 14 predictors were selected (Additional file 1: Appendix S1, Table S4) and used in each classifier to predict AKI. In the internal validation set, GBM model achieved the greatest AUC (0.76, CI 0.70 to 0.82), a highest F1-score (0.73, CI 0.66 to 0.78) that tied with ADA, and relatively balanced sensitivity (0.74, CI 0.66 to 0.8) and specificity (0.65, CI 0.55 to 0.73) (Fig. 2). Since GBM algorithm is more robust to outliers compared to ADA, we eventually chose GBM model for further analysis and application.

Since Kalisvaart’s AKI prediction score was built upon exclusion of patients requiring preoperative CRRT [5], we validated and compared the performance of this score and our GBM-based predictor in the complete internal validation set first, then further compared them in a subset excluding patients that received preoperative CRRT. It turned out that the AKI prediction score presented in our internal validation set an absolutely high specificity (1.0, CI 1.0 to 1.0) with the lowest AUC (0.52, CI 0.45 to 0.6), F1-score (0.03, CI 0.0 to 0.08) and sensitivity (0.02, CI 0.00 to 0.04). These metrics were not improved even in the subset excluding patients receiving preoperative CRRT. Meanwhile, GBM model also demonstrated higher AUC (0.74, CI 0.67 to 0.8), acceptable specificity (0.68, CI 0.59 to 0.77) and sensitivity (0.64, CI 0.56 to 0.73) after exclusion of patients requiring pre-LT dialysis.

### Temporal external validation

**Fig. 3:**
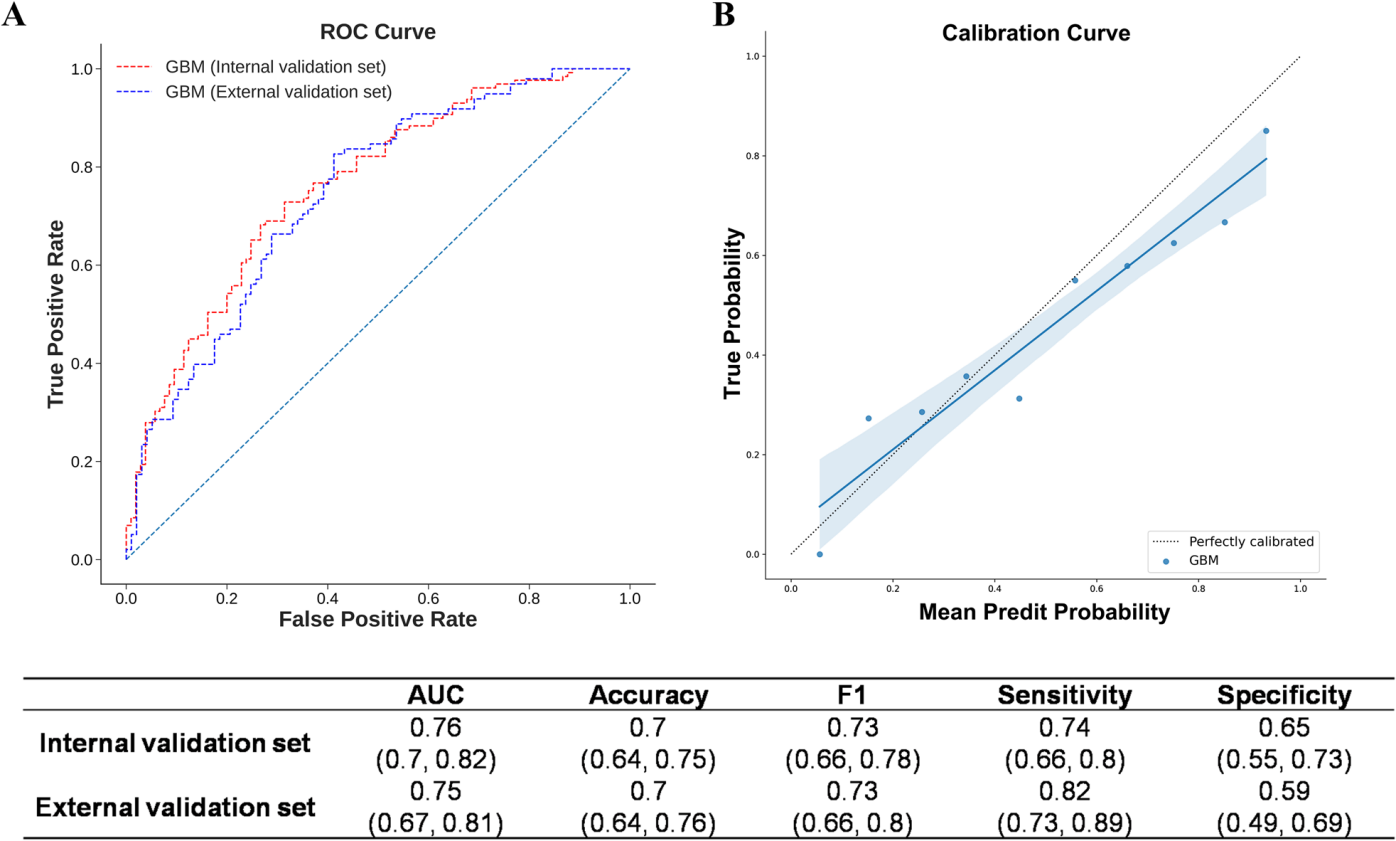
Performance of external validation. **A** Performance of GBM model on the internal validation set and on the external validation set. **B** Calibration plot of current external validation

The external validation set also consisted of a majority of male (87.69%) with a mean age of 47 years old (Table 2). The percentage of graft steatosis graded NASH CRN 1 or above was significantly higher in the external validation set (43.59% vs 26.92%, *p* = 0.001) compared to that of the development set. On the other hand, time under general anesthesia, estimated blood loss, use of colloid and cryoprecipitate were significantly lower in the external validation set. In this temporal validation set, the incidence of AKI was 50.26%, and GBM model achieved a comparable AUC (0.75, CI 0.67 to 0.81) to that of the internal validation set (Fig. 3).

**Table 2:** Comparison of development set and the temporal validation set

Characteristics	Development set (n = 546)	Temporal validation set (n = 195)	P values
Diagnosis of post-LT AKI	301 (55.13%)	98 (50.26%)	0.867
Demographics			
Gender (male, n)	472 (86.45%)	171 (87.69%)	1
Age (y)	50.61 (10.76)	47.02 (10.07)	< 0.001
Height (cm)	167.77 (9.55)	168.55 (6.42)	0.292
Weight (kg)	64.25 (11.42)	65.13 (11.14)	0.35
BMI	22.71 (3.33)	23.09 (3.06)	0.164
Predicting variables			
IBIL (μmol/L)	90.34 (97.04)	96.91 (109.27)	0.433
UO (ml/(kg*h))	3.09 (2.2)	3.03 (1.99)	0.73
Time under GA(min)	543.0 (121.0)	498.86 (111.18)	< 0.001
PLT(10^9^/L)	94.45 (80.83)	93.89 (76.62)	0.932
Steatosis grade ≥ 1	147 (26.92%)	85 (43.59%)	0.001
Preoperative LOS (d)	18.23 (21.82)	15.78 (21.13)	0.175
EBL (ml)	2066.38 (1906.18)	1559.1 (1918.04)	0.002
ALB (g/L)	35.56 (4.89)	34.74 (6.96)	0.133
Bicarbonate (ml)	124.04 (211.47)	169.92 (203.77)	0.009
Colloid (ml)	111.24 (301.53)	32.31 (117.68)	< 0.001
Pre-operative HE (n)	129 (23.63%)	37 (18.97%)	0.899
Cryoprecipitate(U)	30.46 (16.03)	26.53 (15.13)	0.003
ALT (U/L)	131.08 (433.19)	72.26 (211.4)	0.069
Pre-operative HM (n)	209 (38.28%)	91 (46.67%)	0.249

AKI, acute kidney injury’; IBIL, indirect bilirubin; UO, urine output; GA, general anesthesia; PLT, platelets; LOS, length of stay; EBL, estimated blood loss; ALB, albumin; HE, hepatic encephalopathy; ALT, alanine transaminase; HM, hepatic malignancy

### Feature importance evaluated by SHAP values

**Fig. 4:**
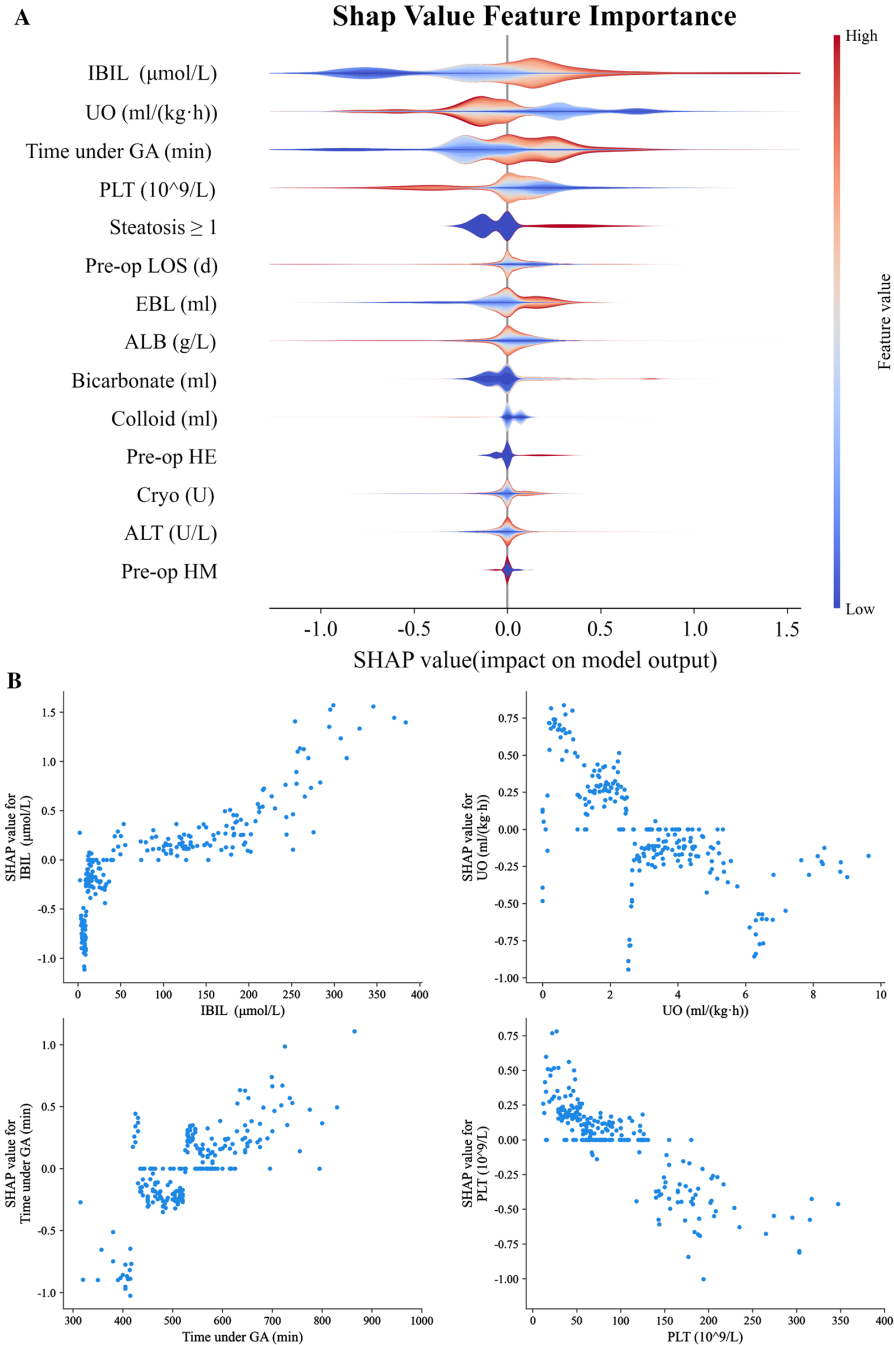
SHAP summary plot and dependence plot. **A** The SHAP summary plot demonstrated the general importance of each feature in GBM model. The color bar on the right indicates the relative value of a feature in each case. Red dots indicate high values and blue dots indicate low values. The violin graph lining up on the midline is the aggregation of dots representing each case in the internal validation set. The distance between the upper and lower margin of the violin graph represents the amount of the cases that end up with the same SHAP values offered by this feature. Categorical features including preoperative HE and HM and steatosis ≥ 1 were represented by 0 and 1, while “0” means “No” and “1” means “Yes”. **B** SHAP dependence plot demonstrated the distribution of SHAP output value of a single feature. In our GBM prediction model, higher IBIL, lower intraoperative urine output, longer time under anesthesia and lower preoperative PLT are correlated with higher SHAP values, representing higher probability of a prediction that favors the diagnosis of AKI

The baseline for the Shapley value in our study is the average of all predicted AKI incidence in the internal validation set, which was 52.08%. In our internal validation set with 234 cases, 163 cases were correctly classified. The SHAP summary plot demonstrated that preoperative IBIL, intraoperative urine output, time under general anesthesia, preoperative PLT and graft steatosis ranked the top 5 important features (Fig. 4A). Both kinds of SHAP plot revealed that higher IBIL, lower urine output, lower PLT, longer anesthesia time and graft steatosis above NASH CRN 1 were associated with higher SHAP value output in GBM model, indicating higher probability of post-LT AKI (Fig. 4). The SHAP summary plot of the rest of the four ML models also demonstrated that IBIl and urine output ranked among the top 3 important features respectively in each model (Additional file 2: Appendix S2, Figure S2).

**Fig. 5:**
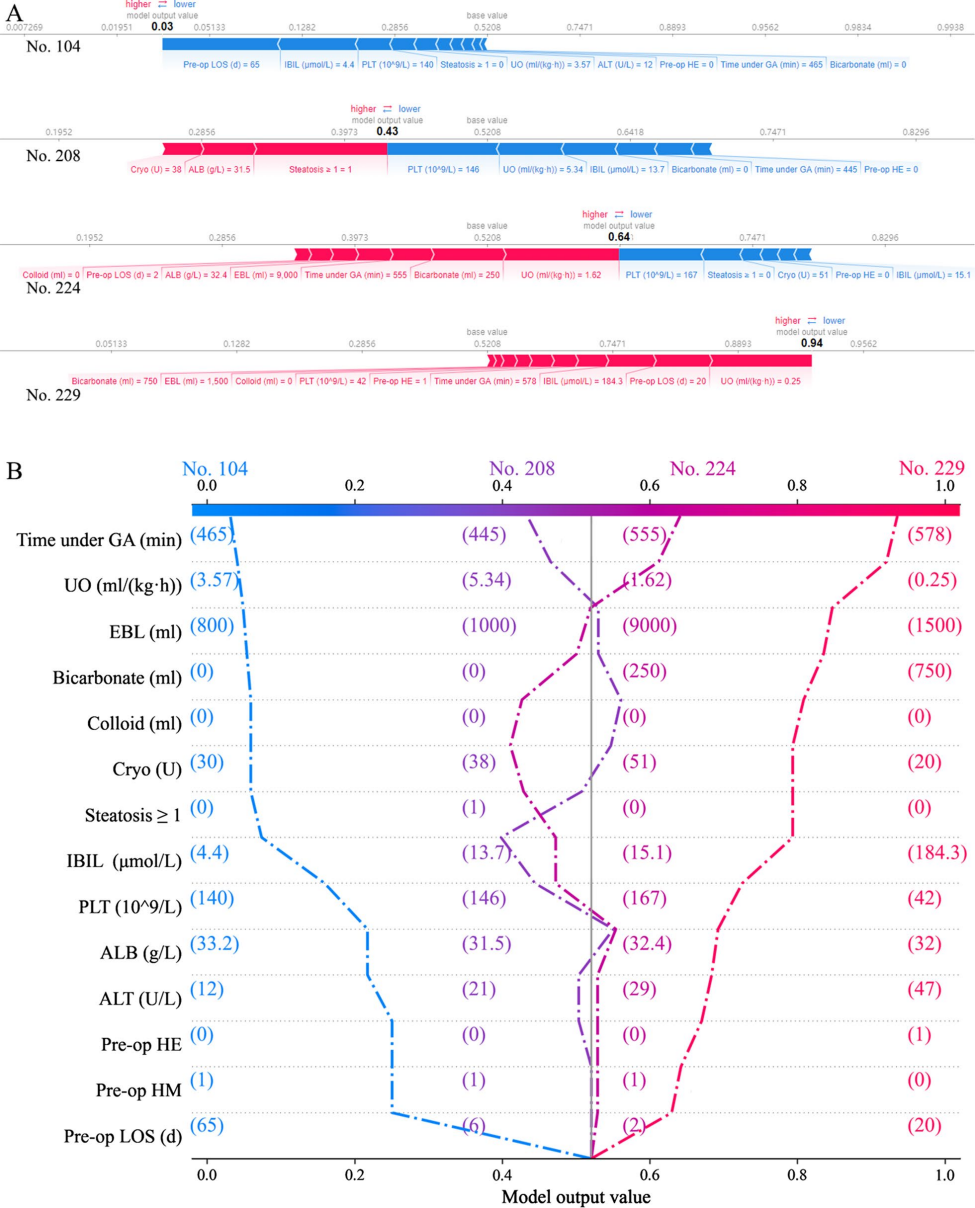
SHAP decision plot and force plot. **A** SHAP force plots of 4 examples of patients, including patient No. 104, No 208, No. 224 and No.229. The features shown in red push the AKI probability towards the right, while the features shown in blue push the probability towards the left. This plot helps physicians to identify easily the major features with high decision power in the model on individual level. **B** SHAP decision plot of the 4 patients in A. This plot is a better visualization of the feature importance of all predictors in each individual. The decision path tended to make drastic turns at feature with high importance and reached the estimated probability of AKI. Physicians can interpret the navigation made by the features and make a personal decision on the credibility of the output

**Fig. 6:**
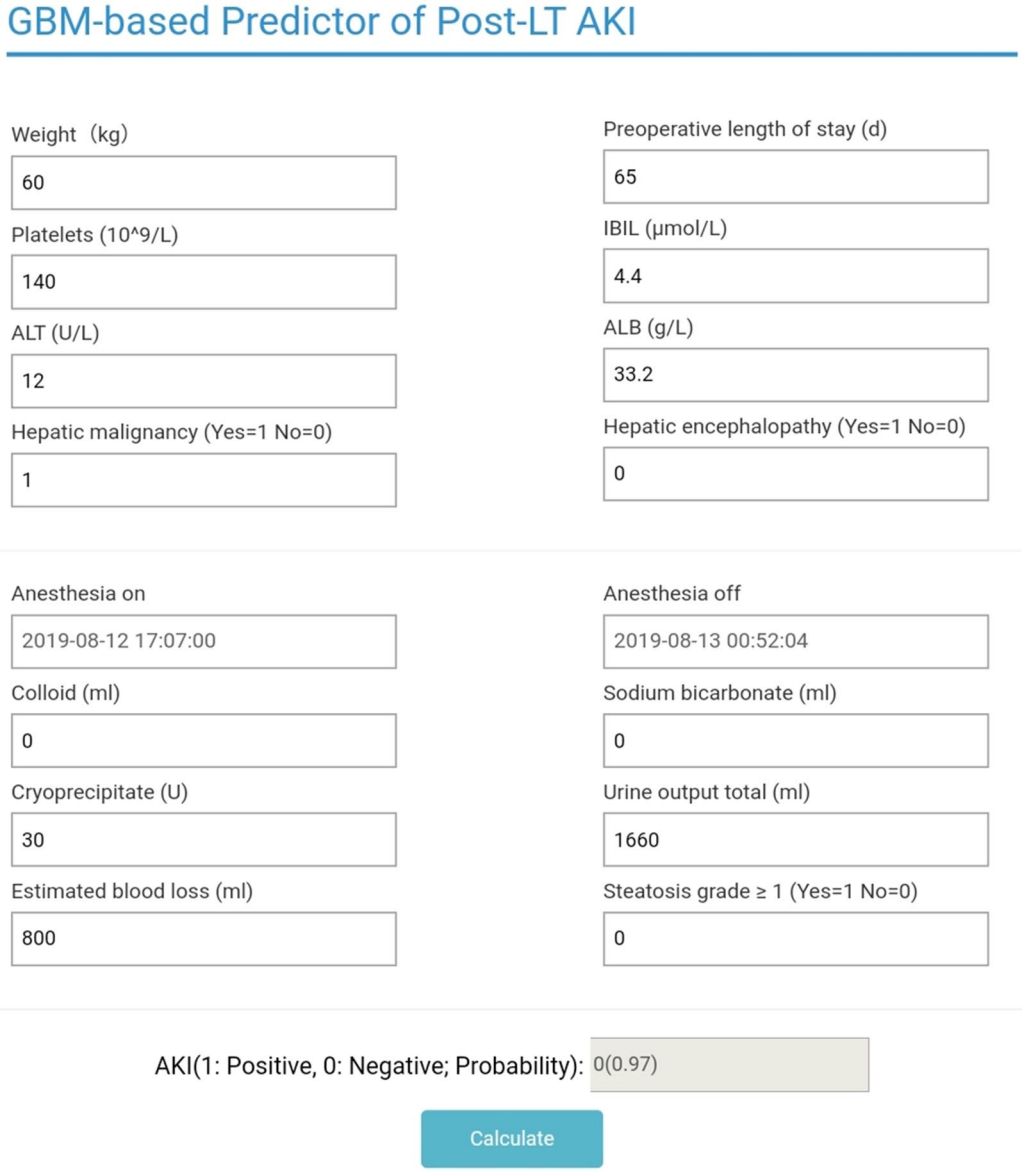
A demo prediction of patient No.104 by online GBM-based predictor of post-LT AKI. A demo prediction of patient No. 104 made by the online GBM-based predictor of post-LT AKI is shown. To increase clinical applicability, intraoperative average urine output and time of anesthesia were substituted by direct input of weight, total urine output and the time of initiation and terminal of anesthesia. The prediction output for patient No. 104 was “0” with a probability of 97%, that is, the probability of this patient developing post-LT AKI was merely 3%

Four examples of correctly classified cases (Patient No. 104, No. 208, No. 224 and No. 229) were demonstrated as SHAP decision plot and force plot in Fig. 5. The SHAP decision plots simulated the path of decision along which each feature was given in a sequence according to their availability in EMRs. The force plot mainly presented the major factors that contribute to the final model output in a certain individual. These plots increased the transparency of the prediction made by GBM algorithm. An online risk calculator to further facilitate external validation can be visited at http://wb.aidcloud.cn/zssy/aki.html (Fig. 6).

## Discussion

### Interpretation

The cause of post-LT AKI is multifaceted. Patients with end-stage liver disease tend to have preoperative intravascular volume depletion and coagulation deficiency that predispose them to greater intraoperative blood loss and low renal perfusion [25]. Besides, the technique of LT involves partial or side cross-clamping of venous flow above the renal vein during anhepatic phase, which contributes to renal congestion and impairs urine output. The 14 predictors incorporated in our model are mainly indicators of preoperative liver dysfunction, intraoperative volume depletion, graft quality and difficulty of the surgery, which were carefully selected by univariate test and subsequent LASSO regression analysis from a series of variables that had been documented as potential risk factors associated with AKI. Moreover, their correlation with AKI were further demonstrated by SHAP summary plot and dependence plot, in which their distribution in relation to the AKI diagnosis were in line with the pathophysiology mentioned above, adding clinical credibility to our model.

We can also tell from these correlations uncovered by ML algorithm that optimization of potentially modifiable variables exerting high importance in predicting AKI, such as intraoperative urine output, preoperative PLT and time under anesthesia, should be given higher priority pre- and intra-operatively. For instance, higher sentinel level of urine output might be considered in patients receiving LT. As has been shown in the SHAP dependence plot, SHAP values distribution tend to be divided around an average urine output of 2.2 ml/(kg·h), which indicates that this might be a potential threshold for physicians to intervene. On the other hand, the criteria in KDIGO guideline requires merely an urine output below 0.5 ml/(kg·h) for at least 6 h to diagnose AKI. Although we did not use this criteria in our research since serum SCr was a more sensitive biomarker to diagnose post-LT AKI in the regimen we adopted, the correlation recognized by ML algorithms illuminate that a higher cut-off point of intraoperative urine output may serve to remind the physicians of renal-protective intervention in advance.

Similarly, our results also indicate that higher PLT transfusion threshold and early extubation shall be preferred in patients receiving LT. Moreover, while graft steatosis of NASH CRN 1 (steatosis involving 5% to 33% of hepatocytes) is accepted in non-urgent LT due to worldwide scarcity of organ donation, it has been identified as a risk predictor of moderate importance by ML algorithms. More strict preliminary graft assessment or lower tolerance in steatosis threshold may be evaluated in the upcoming studies.

Attempts to predict AKI after LT have been made by implementing either novel ML algorithms or conventional statistical technique [5, 6, 9], yet one commonly recognized state-of-the-art prediction system specifically for post-LT AKI setting is currently lacking. Lee, H et al. used a total of 72 pre- and intra-operative variables and also demonstrated that GBM-based model showed best statistical performance to predict post-LT AKI [9]. Nevertheless, the disparities in techniques like use of venovenous bypass and femoral artery pressure make it hard to use our data set to externally validate this model. Yin Z. et al. identified that CIT (> 7 h), donor WIT (> 10 min), blood loss (> 2500 ml), SCr (> 354 μmol/L), treatment period with dopamine (> 6 days) and overexposure to calcineurin inhibitor (CNI) may be potential risk factors of AKI in Chinese liver transplantation cohort [6]. Nevertheless, in our cohort we discovered that the majority of post-LT AKI cases were diagnosed during the first 24 h postoperatively even with delayed Tacrolimus introduction. Meanwhile, a growing proportion of DBD donors without donor WIT has altered the graft characteristics of the cohort. Therefore the power in risk stratification of these factors should be reconsidered and re-analyzed.

Finally we decided to use Kalisvaart’ s AKI prediction score as a benchmark because of our similarity in statistical performance and immunosuppression therapy [5]. As a result, our GBM-based predictor demonstrated higher AUC and F1-score compared to AKI prediction score, either in our original internal validation set or the subset conforming to their criteria that excluded patients requiring preoperative CRRT. We agreed to include patients with preoperative renal injury because these patients have a high possibility of renal recovery after transplantation [20], and are likely to be elevated in the waiting list. Early identification of deterioration in renal function in these patients would be of greater value compared to patients without preoperative renal injury. Considering the preciousness of liver graft and detrimental outcomes associated with AKI, we valued model sensitivity, that is, the ability to find out as much as possible the occurrence of AKI, over model specificity. Comparing to other ML models, boosting algorithms like GBM and ADA achieved generally highest precision and sensitivity, which is consistent with their performance of other studies [26, 27].

## Limitations

One limitation of the current study is that it is a single center study. Liver transplantation is a highly specialized and complicated technique. Only by joint effort made by multiple centers can we build a larger data set. However, multi-center validation calls for unification in feature availability and standardized perioperative treatment. Nevertheless, we utilized the data of a temporally independent cohort to validate our model. Temporal validation is a type of external validation in which data of new cases, though are from the same institution as in the development sample, come in a different (preferably later) time period. And it is considered to be a kind of arguable but acceptable external validation in the TRIPOD statement (Type2b), an intermediary between internal and external validation [19]. It was worth noting that our development set and the temporal validation set demonstrated a bit of heterogeneity in several predictors, such as steatosis grade of donor liver, time under general anesthesia, estimated blood loss, use of colloid, bicarbonate and cryoprecipitate. These changes mainly arose from the improvement of surgical techniques and aggravated scarcity of non-steatotic donors. The incidence of AKI tended to be lower but the drop was not significant. We believe that these significant differences to some extent reflect the effectiveness of our temporal external validation result, as well as the robustness of our model. On the other hand, as for geographical external validation, the features utilized in our model are all regularly recorded or tested in OLT cases in most transplant centers, and multicenter cooperation can be achieved once authorization of data usage is approved.

Another possible limitation is that the statistical metrics of our model might not be as high as those presented in similar researches [9, 28]. However, many of these studies built their ML models upon high dimensional features, running the risk of over-fitting. After careful feature elimination, we built our predicting model with merely 14 features, aiming for practical external validation in the future. In this way it was worthy trading statistical accuracy for model applicability. Moreover, the path of decision made by our model in each individual can be illustrated as SHAP decision plot, offering richer information in feature importance or even in potential drawbacks of the model. With such visualized explanation, physicians can interpret the model output easily and timely adjust their decisions.

### Implications

Our research is a solid and generalizable work to build an applicable predictor of post-LT AKI with supervised ML, which covers the prediction of AKI in patients requiring preoperative renal replacement therapy. The GBM-based model we developed consists of variables with high clinical credibility that are interoperable across institutions, and demonstrates satisfactory statistical validity and reasonable relational interpretability revealed by SHAP method.

As an emerging tool of explanatory AI, SHAP method can facilitate both local and global interpretations [12, 29]. For local interpretation, each case has its own set of SHAP values. So it can explain how each feature contributes to the prediction of a certain case, as has been illustrated in our SHAP decision plot and force plot, which increases transparency and helps clinicians analyze the credibility of the prediction model. For global interpretability, the aggregate value of SHAP shows the importance of each predicting variable. Compared with traditional methods to evaluate feature importance such as the weight of RF, the SHAP value holds better consistency and can present the positive or negative relationship of each predictor.

The potential application of this model lies in its integration with the EMRs system to guide early diagnosis and interventions after LT. Since the features we selected are all easily accessible right at the end of the surgery, this GBM-based predictor of post-transplant AKI would be a convenient predicting tool that can maintain transparency of the decision-making process to clinical physicians, enabling them to adjust the final decision according to their own experience.

## Supplementary Information

**Additional file 1: Selection and definition of variables. Appendix S1. Table S1.** Definition of special complications or terms. **Table S2.** All of the 111 variables that were chosen for initial selection. **Table S3.** The 38 features selected by univariate test. **Table S4.** The features selected by LASSO regression.

**Additional file 2: Model Development, Validation and SHapley Additive exPlanation. Appendix S2. Table S1.** The best hyperparameters of each classifier. **Table S2.** Comparison between the development set and the internal validation set. **Table S3.** Performance of machine learning models and AKI prediction score. **Table S4** Comparison of performance between GBM model and other models. **Table S5.** Performance of GBM and AKI prediction score in the cohort excluded preoperative CRRT. **Table S6.** Comparison between the development set and external validation set. **Table S7.** Performance of GBM model in the original test set and in the external validation set. **Figure S1.** Predicting performance using the top variables identified by SHAP importance plot. **Figure S2.** SHAP summary plot of 4 machine learning models besides GBM.

**Additional file 3: Complete Statistics. Table S1.** Statistics of the 111 variables that was chosen for initial selection. **Table S2.** Post-operative medications prior to the diagnosis of AKI or prior to the appearance of maximum SCr in Non-AKI group. **Table S3.** Stage and time of diagnosis of AKI. **Table S4** Coefficient in LASSO analysis of the 38 variables selected by univariate test.

**Additional file 4: Anesthesia and Immunosuppression Therapy. Appendix S4.** Anesthesia and Immunotherapy.

**Additional file 5:** Kidney Disease Improving Global Outcomes (KDIGO) diagnostic criteria of AKI.

**Additional file 6: Meld(i) Score. Table S1**. Exceptional conditions to be assigned higher MELD score.

## Data Availability

All the analyzed results during this study are included in the appendices. The datasets analysed during the current study are available from the corresponding author on reasonable request.

## Abbreviations

HCT: Hematocrit
PLT: Platelets
WBC: White blood cells
ALT: Alanine transaminase
AST: Aspartate transaminase
TBIL: Total bilirubin
DBIL: Direct bilirubin
IBIL: Indirect bilirubin
ALB: Albumin
SCr: Serum creatinine
BUN: Blood urea nitrogen
PT: Prothrombin time
APTT: Activated partial thromboplastin time
FIB: Fibrinogen
INR: International normalized ratio
MELD: Model of end-stage liver disease
EBL: Estimated blood loss
CRRT: Continuous renal replacement therapy
AUC: Area under the receiver operating characteristic curve
ROC: Receiver operating characteristic curve
WIT: Warm ischemia time
CIT: Cold ischemia time
LOS: Length of stay
ICU: Intensive care unit
AKI: Acute kidney injury
LT: Liver transplantation
LASSO: Least absolute shrinkage and selection operator
RF: Random forest
LR: Logistic regression
SVM: Support vector machine
GBM: Gradient boosting machine
ADA: Adaptive boosting
DCD: Donation after circulatory death
DBD: Donation after brain death
DBCD: Donation after brain death followed by circulatory death
SHAP: Shapley additive explanations
CKD: Chronic kidney disease
EMRs: Electronic medical records

## Code availability

The codes used in this study are all common codes in Python packages mentioned in the part of “Methods” in the manuscript.
