# Supplementary material for: An explainable supervised machine learning predictor of acute kidney injury after adult deceased donor liver transplantation
Source: J Transl Med. 2021 Jul 28;19:321. doi: 10.1186/s12967-021-02990-4 (PMC8317304; doi:10.1186/s12967-021-02990-4)
Supplement: Supplementary file 1 — Additional file 1: Selection and definition of variables. Appendix S1. Table S1. Definition of special complications or terms. Table S2. All of the 111 variables that were chosen for initial selection. Table S3. The 38 features selected by univariate test. Table S4. The features selected by LASSO regression. [file 12967_2021_2990_MOESM1_ESM.docx]

**Appendix S1: Selection and definition of variables**

**Definition of Variables**

A database platform named Perioperative Period Database Platform (PPDP) with an integrated Nature Language Processing (NLP) module was applied to extract integrated medical data generated during admission. Most variables were directly extracted from electronic medical records (EMRS) from hospital information system, laboratory information system, picture archiving and communication system and Docare Anesthesia System (2005-2020 Medical system Co., Ltd. Suzhou, China). To be specific, the keywords designating target medical terms involving nomenclature of diseases, comorbidities, complications, treatment and medications were manually annotated on 10% of the text in EMRs. Then the NLP module could automatically extract the structured information from the remaining 90% EMR text with an accuracy over 98%. Variables listed in Table S1 were collected by applying special rules when searching through EMRs. Variables concerning donor characteristics were collected and anonymized by one surgeon in the Department of Liver Transplant of our hospital who has registered access to China Organ Transplant Response System.

**Table S1 Definition of special complications or terms**

| **Variables** | **Definition** |
| --- | --- |
| Preoperative ARDS | ARDS was diagnosed when all of the following conditions were met:  1.Preoperative PaO_2_/FiO_2_ ratio below 200;  2.Preoperative pulmonary infiltrates demonstrated by chest X-rays or thoracic spiral CT;  3. The term “ARDS” was extracted by NLP module from admission diagnosis. |
| Preoperative ALI | ALI was diagnosed when all of the following conditions were met:  1. Preoperative PaO_2_/FiO_2_ ratio below 300;  2.Preoperative pulmonary infiltrates demonstrated by chest X-rays or thoracic spiral CT. |
| Preoperative AKI | The ratio of preoperative maximum SCr to minimum SCr was over 1.5. |
| Preoperative CKD | Identified eGFR below 60 for 3 months or more or identified markedly high levels of albumin in urine test |
| Recipient warm ischemia time | The time of liver implantation, from removal of the organ from ice until reperfusion. (This is also the definition used in the work of Kalisvaart et al.). |
| Hypokalemia | Serum potassium was lower than 3.5 mmol/L. |
| Hyperkalemia | Serum potassium was higher than 5.5 mmol/L. |
| Hyponatremia | Serum sodium was lower than 135 mmol/L. |
| Hypernatremia | Serum sodium was higher than 145 mmol/L. |
| Hypocalcemia | Ionized calcium was lower than 1.0 mmol/L. |
| Hypercalcemia | Ionized calcium was higher than 2.75 mmol/L. |
| Metabolic acidosis | HCO_3_^-^ was below 20.2 mmol/L. |
| Arrhythmia | Arrhythmia was diagnosed when any of the criteria was met:  1. Detection of any intraoperative heart rate over 100 bpm or below 50 bpm;  2. Intraoperative use of any of the following antiarrhythmic drugs including amiodarone, lidocaine, lanatoside D, epinephrine, atropine or isoprenaline was identified. |
| Cardiac arrest | The term “cardiac arrest” was identified by NLP module from surgical or anesthesia record. |
| Acidosis | pH in blood gas result was lower than 7.30. |
| Hyperlactacidemia | Serum lactate was over 2.0 mmol/L. |
| Intraoperative hypotension | Hypotension was diagnosed when any intraoperative invasive or non-invasive systolic pressure below 90 mmHg or any invasive or non-invasive mean arterial pressure was below 60 mmHg. |
| SCr_Mean | The mean of the maximum value and the minimum value of preoperative SCr. |

ARDS = acute respiratory distress syndrome; ALI = acute lung injury; AKI = acute kidney injury; SCr = serum creatinine; CKD = chronic kidney disease.

**Selection of Features**

**A total of 111potentially relevant variables generated out of empirical experience and literature review were collected for initial analysis (Table S2).** The results of the latest laboratory test prior to surgery were collected. The doses of intraoperative bolus medication collected were the accumulative sum by the end of the surgery. Since the missing proportion of height was greater than 10%, we excluded “Height” “Weight” and “Body mass index”. To avoid multicollinearity, we excluded “TBIL” (the sum of DBIL and IBIL) and “MELD score” (which was calculated by TBIL, INR and SCr with special rules adding points to patients with hepatic malignancy).

To minimize potential overfitting brought by high dimensionality of the features, we first selected features that were statistically significant in univariate test. To make our predictor more clinically applicable, we transform the feature “Steatosis of donor liver” into 2 new features, “Steatosis grade ≥ 1” and “Steatosis grade ≥ 2”, both of which turned out to be statistically significant in univariate test as well. Finally, 38 variables (Table S3) were subjected to a least absolute shrinkage and selection operator (LASSO) regression to select features with a coefficient above zero. 17 features were chosen as predictors for further model construction (Table S4). Since the availability and indication for recombinant activator Factor VII (rFVIIa), terlipressin and prothrombin complex concentrate may differ greatly across transplant centers, we excluded these features as predictors to ensure the interoperability of our predicting model among institutions. Finally, 14 features were subjected to model building.

**Table S2 All of the 111 variables that were chosen for initial selection**

| **Demographics** | **Preoperative complications** | **Surgery characteristics** |
| --- | --- | --- |
| Gender (male, n) | MELD score | Time of surgery(min) |
| Age (y) | Portal hypertension (n) | Time under GA (min) |
| Height (cm) | Ascites (n) | Recipient warm ischemic time (min) |
| Weight (kg) | Acute kidney injury (n) | Cold ischemic time (h) |
| Body Mass Index | Hepatorenal syndrome (n) | Complicated hepatic artery reconstruction (n) |
| Preoperative LOS (d) | CRRT (n) | Choledochojejunostomy (n) |
|  | Frequency of CRRT (times) | Surgical technique |
| **Preoperative comorbidities** | Hepatic encephalopathy (n) |  |
| Hypertension (n) | Plasmapheresis (n) | **Intraoperative fluid and transfusion** |
| Diabetes mellitus (n) | Hepato-pulmonary syndrome(n) | Crystalloid (ml) |
| Myocardial infarction (n) | ARDS (n) | Colloid (ml) |
| Coronary artery disease (n) | Acute lung injury (n) | Albumin (ml) |
| Chronic kidney disease (n) | Mechanical ventilation (n) | Other fluids (ml) |
| Smoking (n) | ICU stay (n) | RBC (ml) |
| Alcohol abuse (n) | Hypokalemia (n) | Plasma (ml) |
| Previous surgery (n) | Hyperkalemia (n) | Cryoprecipitate (U) |
|  | Hyponatremia (n) | EBL (ml) |
| **Donor characteristics** | Hypernatremia (n) | Urine output (ml/(kg·h)) |
| Donor age (y) | Hypocalcemia (n) | Ascites removal (ml) |
| Donor BMI | Hypercalcemia (n) | Gastric drainage (ml) |
| ABO incompatibility (n) | Metabolic acidosis (n) | Other estimated fluid loss (ml) |
| Donor Type |  |  |
| Steatosis of donor liver | **Preoperative laboratory values** | **Intraoperative medication** |
|  | HCT | rFVIIa (mg) |
| **Etiology of liver** | PLT(10^9/L) | Prothrombin complex concentrate (IU) |
| Hepatitis B (n) | WBC (10^9/L) | Fibrinogen (g) |
| Hepatitis C (n) | ALT (U/L) | Terlipressin (mg) |
| Dual infection (n) | AST (U/L) | Norepinephrine, bolus (mg) |
| Hepatic malignancy (n) | TBIL (μmol/L) | Epinephrine, bolus (mg) |
| Drug-induced liver injury (n) | DBIL (μmol/L) | Dopamine, bolus (mg) |
| Alcohol-related liver disease (n) | IBIL (μmol/L) | Bicarbonate (ml) |
| Auto-immune hepatitis（n） | ALB (g/L) | Use of norepinephrine, continuous (n) |
| Hepatolenticular degeneration(n) | SCr (μmol/L) | Use of epinephrine, continuous (n) |
| Hemochromatosis (n) | BUN (mmol/L) | Use of dopamine, continuous (n) |
| Cirrhosis (n) | PT (s) | Use of aramine (n) |
| Primary biliary cirrhosis (n) | APTT (s) |  |
| Alcoholic liver cirrhosis (n) | FIB (g/L) | **Intraoperative incident** |
|  | INR | Arrhythmia (n) |
|  | K+ (mmol/L) | Cardiac arrest (n) |
|  | Na+ (mmol/L) | Acidosis (n) |
|  | Ca++(mmol/L) | Hyperlactacidemia (n) |
|  | HCO3- (mmol/L) | Hypokalemia (n) |
|  | eGFR (ml/(min·1.73^2)) | Hypernatronemia (n) |
|  | SCr_Mean (μmol/L) | Hypotension (n) |
|  |  |  |

BMI = body mass index; LOS = length of stay; MELD = model for end stage liver disease. CRRT = continuous renal replacement therapy; ARDS = acute respiratory distress syndrome; ICU = intensive care unit; HCT = hematocrit; PLT = platelets; WBC = white blood cell; ALT = alanine transaminase; AST = aspartate transaminase; TBIL = total bilirubin; DBIL = direct bilirubin; IBIL = indirect bilirubin; ALB = albumin; SCr = serum creatinine; BUN = blood urea nitrogen; PT = prothrombin time; APTT = activated partial thromboplastin time; FIB = fibrinogen; INR = international normalized ratio; eGFR = estimated glomerular filtration rate; GA = general anesthesia; RBC = red blood cell; EBL = estimated blood loss; rFVIIa = recombinant activated factor VII.

**Table S3 The 38 features selected by univariate test**

| **Preoperative features** | **Intraoperative features** |
| --- | --- |
| Preoperative LOS (d) | ABO incompatibility (n) |
| HCT | Steatosis grade ≥ 1 |
| PLT(10^9/L) | Steatosis grade ≥ 2 |
| ALT (U/L) | Time of surgery(min) |
| AST (U/L) | Time under GA(min) |
| DBIL (μmol/L) | Colloid (ml) |
| IBIL (μmol/L) | RBC (ml) |
| ALB (g/L) | Cryoprecipitate (U) |
| PT (s) | EBL (ml) |
| APTT (s) | Urine output (ml/(kg*h)) |
| FIB (g/L) | rFVIIa (mg) |
| INR | Prothrombin complex concentrate (IU) |
| Hepatic malignancy (n) | Terlipressin (mg) |
| Cirrhosis (n) | Bicarbonate (ml) |
| Hepatorenal syndrome (n) | Use of aramine (n) |
| CRRT (n) | Cardiac arrest (n) |
| Frequency of CRRT (times) |  |
| Hepatic encephalopathy(n) |  |
| Mechanical ventilation (n) |  |
| ICU stay (n) |  |
| Hypernatremia (n) |  |
| Hypercalcemia (n) |  |

LOS = length of stay; ICU = intensive care unit; HCT = hematocrit; CRRT = continuous renal replacement therapy; PLT = platelets; ALT = alanine transaminase; AST = aspartate transaminase; DBIL = direct bilirubin; IBIL = indirect bilirubin; ALB = albumin; PT = prothrombin time; APTT = activated partial thromboplastin time; FIB = fibrinogen; INR = international normalized ratio; GA = general anesthesia; RBC = red blood cell; EBL = estimated blood loss; rFVIIa = recombinant activated factor VII.

**Table S4 The features selected by LASSO regression**

| Preoperative liver dysfunction | Intraoperative volume depletion |  | Graft quality |  | Difficulty of surgery |
| --- | --- | --- | --- | --- | --- |
| Preoperative LOS (d) | Colloid (ml) |  | Steatosis grade ≥ 1 |  | Time under GA (min) |
| PLT (10^9/L) | Cryoprecipitate (U) |  |  |  | Bicarbonate (ml) |
| ALT (U/L) | EBL (ml) |  |  |  |  |
| IBIL (μmol/L) | rFVIIa (mg) |  |  |  |  |
| ALB (g/L) | Terlipressin (mg) |  |  |  |  |
| Hepatic malignancy (n) | Prothrombin complex concentrate (IU) |  |  |  |  |
| Hepatic encephalopathy (n) | Urine output (ml/(kg·h)) |  |  |  |  |

**To strengthen the interoperability of our predicting model across institutions and facilitate further external validation, medications like rFVIIa, terlipressin, and prothrombin complex concentrates were excluded, leaving only 14 predictors for model building.** LASSO = least absolute shrinkage and selection operator; LOS = length of stay; PLT = platelets; ALT = alanine transaminase; IBIL = indirect bilirubin; ALB = albumin; EBL = estimated blood loss; rFVIIa = recombinant activated factor VII; GA = general anesthesia.
