# Supplementary material for: An explainable supervised machine learning predictor of acute kidney injury after adult deceased donor liver transplantation
Source: J Transl Med. 2021 Jul 28;19:321. doi: 10.1186/s12967-021-02990-4 (PMC8317304; doi:10.1186/s12967-021-02990-4)
Supplement: Supplementary file 2 — Additional file 2: Model Development, Validation and SHapley Additive exPlanation. Appendix S2. Table S1. The best hyperparameters of each classifier. Table S2. Comparison between the development set and the internal validation set. Table S3. Performance of machine learning models and AKI prediction score. Table S4 Comparison of performance between GBM model and other models. Table S5. Performance of GBM and AKI prediction score in the cohort excluded preoperative CRRT. Table S6. Comparison between the development set and external validation set. Table S7. Performance of GBM model in the original test set and in the external validation set. Figure S1. Predicting performance using the top variables identified by SHAP importance plot. Figure S2. SHAP summary plot of 4 machine learning models besides GBM. [file 12967_2021_2990_MOESM2_ESM.docx]

**Appendix S2: Model Development, Validation and SHapley Additive exPlanation**

**Model development and validation**

**Table S1 The best hyperparameters of each classifier**

|  | **Hyperparameters Set** | **Optimal Hyperparameters** | **AUC** |
| --- | --- | --- | --- |
| **LR** | L1 Regularization term: [0.0001, 0.0008, 0.006, 0.046,0.36, 2.78, 21.54, 168.81,1291.54, 10000]  (Smaller value specifies greater regularization strength) | L1 Regularization term: 0.006 | 0.73 |
| **SVM** | Regularization term: {0.1,1,10,20};  Gamma {0.1,1,10,20} | Kernel: Radial Basis  Regularization term: 3  Gamma: 0.01 | 0.75 |
|  |  |  |  |
| **RF** | Number of estimators {10,50,100,500};  Maximum features {3,4,5,6} | Number of estimators: 500  Maximum features: 2  Criteria: Gini | 0.75 |
|  |  |  |  |
| **ADA** | Number of estimators {10,100,500};  Learning Rate: {0.005,0.05,0.01} | Number of estimators: 100  Learning rate: 0.01 | 0.75 |
| **GBM** | Number of estimators: {10,50,100,500};  Learning rate: {0.01,0.05,0.1} | Number of estimators: 100  Learning rate: 0.1 | 0.76 |
|  |  |  |  |

LR = logistic regression; SVM = support vector machine; RF = random forest; ADA = adaptive boosting; GBM = gradient boosting machine implemented by decision tree.

**AKI prediction score**

Kalisvaart M. et al. developed an AKI prediction score according to the Framingham risk scheme. We used the methods offered in their thesis to calculate this score ([1](#_ENREF_1)).

**Table S2 Comparison between the development set and the internal validation set**

| **Characteristics** | **Development set**  **(n = 546)** | **Internal validation set**  **(n = 234)** | **P value** |
| --- | --- | --- | --- |
| Diagnosis of AKI | 301(55.13%) | 129(55.13%) | 1 |
| Predicting variables |  |  |  |
| IBIL (μmol/L) | 90.34(97.04) | 91.0(95.51) | 0.93 |
| UO (ml/(kg*h)) | 3.09(2.2) | 3.14(2.01) | 0.774 |
| Time under GA(min) | 543.0(121.0) | 541.04(126.97) | 0.838 |
| PLT(10^9/L) | 94.45(80.83) | 99.69(76.01) | 0.399 |
| Steatosis grade ≥ 1 | 147(26.92%) | 59(25.21%) | 1 |
| Preoperative LOS (d) | 18.23(21.82) | 17.21(21.18) | 0.545 |
| EBL (ml) | 2066.68(1906.18) | 2016.45(2285.95) | 0.751 |
| ALB (g/L) | 35.56(4.89) | 35.92(4.94) | 0.341 |
| Bicarbonate (ml) | 124.04(211.47) | 133.93(280.73) | 0.589 |
| Colloid (ml) | 111.14(301.5) | 154.6(629.12) | 0.193 |
| Pre-operative HE (n) | 129(23.63%) | 51(21.79%) | 1 |
| Cryoprecipitate(U) | 30.42(15.98) | 30.2(15.48) | 0.853 |
| ALT (U/L) | 131.08(433.19) | 115.08(308.83) | 0.609 |
| Pre-operative HM (n) | 209(38.28%) | 103(44.02%) | 0.633 |

AKI = acute kidney injury; IBIL = indirect bilirubin; UO = urine output; GA = general anesthesia; PLT = platelets; LOS = length of stay; EBL = estimated blood loss; ALB = albumin; HE= hepatic encephalopathy; ALT = alanine transaminase; HM = hepatic malignancy.

**Table S3 Performance of machine learning models and AKI prediction score**

| **Model** | **AUC** | **Accuracy** | **F1** | **Sensitivity** | **Specificity** |
| --- | --- | --- | --- | --- | --- |
| **AKI Prediction Score** | 0.52(0.45, 0.6) | 0.46(0.4, 0.52) | 0.03(0.0, 0.08) | 0.02(0.0, 0.04) | 1.0(1.0, 1.0) |
| **LR** | 0.73(0.66, 0.79) | 0.65(0.59, 0.71) | 0.66(0.58, 0.72) | 0.61(0.52, 0.69) | 0.7(0.61, 0.79) |
| **SVM** | 0.75(0.68, 0.81) | 0.68(0.62, 0.74) | 0.68(0.61, 0.74) | 0.62(0.54, 0.71) | 0.74(0.66, 0.82) |
| **RF** | 0.75(0.68, 0.81) | 0.66(0.6, 0.71) | 0.69(0.62, 0.75) | 0.7(0.62, 0.77) | 0.61(0.51, 0.7) |
| **ADA** | 0.75(0.69, 0.81) | 0.7(0.64, 0.76) | 0.73(0.67, 0.79) | 0.74(0.67, 0.82) | 0.65(0.55, 0.73) |
| **GBM** | 0.76(0.7, 0.82) | 0.7(0.64, 0.75) | 0.73(0.66, 0.78) | 0.74(0.66, 0.8) | 0.65(0.55, 0.73) |

LR = logistic regression; SVM = support vector machine; RF = random forest; ADA = adaptive boosting; GBM = gradient boosting machine implemented by decision tree.

**Table S4 Comparison of performance between GBM model and other models**

| **Model** | **Test** | **AUC** | **Accuracy** | **Sensitivity** | **Specificity** |
| --- | --- | --- | --- | --- | --- |
| **AKI Prediction Score** | T statistics | 153.857 | 170.547 | 581.48 | -234.443 |
|  | P Value | p<0.001 | p<0.001 | p<0.001 | p<0.001 |
| **LR** | T statistics | 21.477 | 33.48 | 71.644 | -28.447 |
|  | P Value | p<0.001 | p<0.001 | p<0.001 | p<0.001 |
| **SVM** | T statistics | 8.424 | 14.974 | 64.541 | -47.438 |
|  | P Value | p<0.001 | p<0.001 | p<0.001 | p<0.001 |
| **RF** | T statistics | 8.491 | 28.232 | 22.205 | 17.833 |
|  | P Value | p<0.001 | p<0.001 | p<0.001 | p<0.001 |
| **ADA** | T statistics | 6.055 | -3.147 | -4.975 | 0.4 |
|  | P Value | p<0.001 | 0.002 | p<0.001 | 0.689 |

LR = logistic regression; SVM = support vector machine; RF = random forest; ADA = adaptive boosting; GBM = gradient boosting machine implemented by decision tree.

**Table S5 Performance of GBM and AKI prediction score in the cohort excluded preoperative CRRT**

| **Model** | **AUC** | **Accuracy** | **F1** | **Sensitivity** | **Specificity** |
| --- | --- | --- | --- | --- | --- |
| **AKI Prediction Score** | 0.52(0.44, 0.59) | 0.47(0.39, 0.54) | 0.03(0.0, 0.09) | 0.02(0.0, 0.05) | 1.0(1.0, 1.0) |
| **GBM** | 0.74(0.67, 0.8) | 0.66(0.59, 0.73) | 0.67(0.6, 0.74) | 0.64(0.56, 0.73) | 0.68(0.59, 0.77) |

According to the work of Kalisvaart M et al., they excluded patients receiving preoperative CRRT in their study. Therefore, we further validated and compared the performance of their AKI prediction score to our GBM model in a subset derived out of our test set that also excluded patients requiring preoperative CRRT. GBM = gradient boosting machine implemented by decision tree.

**Table S6 Comparison between the development set and external validation set**

| **Characteristics** | **Development set**  **(n = 546)** | | **External Validation set**  **(n = 195）** | | **P values** |
| --- | --- | --- | --- | --- | --- |
| Diagnosis of post-LT AKI | | 301(55.13%) | | 98(50.26%) | 0.867 |
| Demographics | |  | |  |  |
| Gender (male, n) | | 472(86.45%) | | 171(87.69%) | 1 |
| Age (y) | | 50.61(10.76) | | 47.02(10.07) | <0.001 |
| Height (cm) | | 167.77(9.55) | | 168.55(6.42) | 0.292 |
| Weight (kg) | | 64.25(11.42) | | 65.13(11.14) | 0.35 |
| BMI | | 22.71(3.33) | | 23.09(3.06) | 0.164 |
| Predicting variables | |  | |  |  |
| IBIL (μmol/L) | | 90.34(97.04) | | 96.91(109.27) | 0.433 |
| UO (ml/(kg*h)) | | 3.09(2.2) | | 3.03(1.99) | 0.73 |
| Time under GA(min) | | 543.0(121.0) | | 498.86(111.18) | <0.001 |
| PLT(10^9/L) | | 94.45(80.83) | | 93.89(76.62) | 0.932 |
| Steatosis grade ≥ 1 | | 147(26.92%) | | 85(43.59%) | 0.001 |
| Preoperative LOS (d) | | 18.23(21.82) | | 15.78(21.13) | 0.175 |
| EBL (ml) | | 2066.38(1906.18) | | 1559.1(1918.04) | 0.002 |
| ALB (g/L) | | 35.56(4.89) | | 34.74(6.96) | 0.133 |
| Bicarbonate (ml) | | 124.04(211.47) | | 169.92(203.77) | 0.009 |
| Colloid (ml) | | 111.24(301.53) | | 32.31(117.68) | <0.001 |
| Pre-operative HE (n) | | 129(23.63%) | | 37(18.97%) | 0.899 |
| Cryoprecipitate(U) | | 30.46(16.03) | | 26.53(15.13) | 0.003 |
| ALT (U/L) | | 131.08(433.19) | | 72.26(211.4) | 0.069 |
| Pre-operative HM (n) | | 209(38.28%) | | 91(46.67%) | 0.249 |

AKI = acute kidney injury; BMI = body mass index; IBIL = indirect bilirubin; UO = urine output; GA = general anesthesia; PLT = platelets; LOS = length of stay; EBL = estimated blood loss; ALB = albumin; HE= hepatic encephalopathy; ALT = alanine transaminase; HM = hepatic malignancy.

**Table S7 Performance of GBM model in the original internal validation set and in the external validation set**

| **Dataset** | **AUC** | **Accuracy** | **F1** | **Sensitivity** | **Specificity** |
| --- | --- | --- | --- | --- | --- |
| **Internal validation set** | 0.76 (0.7, 0.82) | 0.7 (0.64, 0.75) | 0.73 (0.66, 0.78) | 0.74 (0.66, 0.8) | 0.65 (0.55, 0.73) |
| **External validation set** | 0.75 (0.67, 0.81) | 0.7 (0.64, 0.76) | 0.73 (0.66, 0.8) | 0.82 (0.73, 0.89) | 0.59 (0.49, 0.69) |

**Attempts of feature elimination**

We attempted to simplify our model by using only the features of the highest importance to reduce the difficulty of data collection during prospective external validation. However, when we trimmed the predicting features like down to the top 5 variables, the resulting performance was not as satisfactory as the current one. We present here the result of predictive performance by GBM based on top 3, top5 and the current variables.


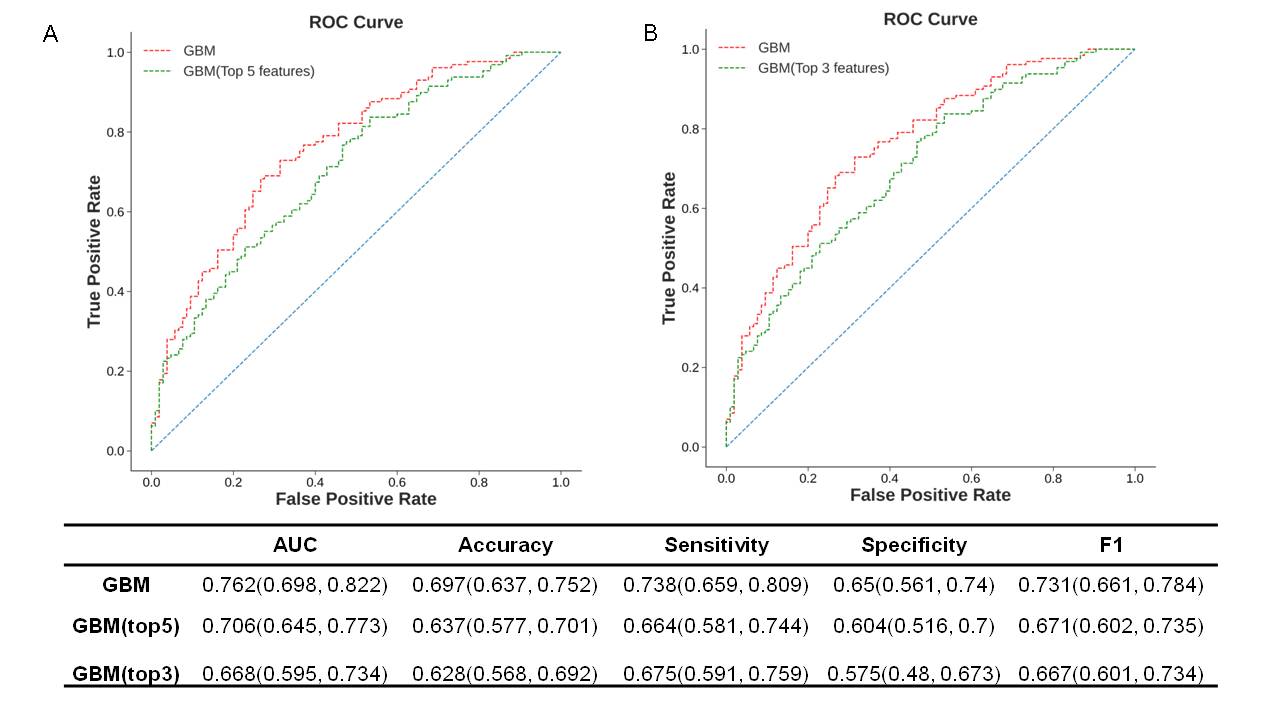


**Figure S1. Predicting performance using the top variables identified by SHAP importance plot. GBM = gradient boosting machine.**

**SHapley Additive exPlanation**

To interpret the predicting process of our GBM-based AKI predictor, the Shapley Additive explanation (SHaP) values were used to provide consistent and locally accurate attribution values for each feature, which inherit simultaneously the black-box local estimation advantages of local interpretable model-agnostic explanations (LIME) and the global consistency of the game theory from Shapley value.

SHAP value can interpret the model prediction by calculating the contribution of each feature to the prediction, that is, the prediction output of weighting and summing all possible feature value combinations (GBM log odds). Such contribution is defined as the Shapley value.

For a model including $p$ features {$x_{1},\ldots, x_{p}\}$, the Shapley value of feature $x_{i}$ can be expressed as a weighted sum for all possible coalitions with weights, i.e.,

$$\phi_{i}(val)=\sum_{S\subseteq F\backslash\{i\}} \frac{\left| S \right|!\left( \left| F \right|-\left| S \right|-1 \right)!}{\left| F \right|!}\left( val(x_{S\cup\left\{ i \right\}})-val\left( x_{S} \right) \right)$$

Where $S$ is a subset of the features used in the model, $F=\{x_{1},\ldots, x_{p}\}$, $x_{i}$ is the vector of feature values of the instance to be explained and $F$ is the number of features, and $val_{x}(S)$ is the prediction for feature values in set $S$ that are marginalized over features that are not included in set $S$:

$$val_{x}\left( S \right)=\int\hat{f}\left( x_{1},\ldots, x_{p} \right)dP_{x\notin S}-E_{X}(\hat{f}(X))$$

In this study, SHAP value was applied on GBM model to reveal feature contribution to model output on individual level.


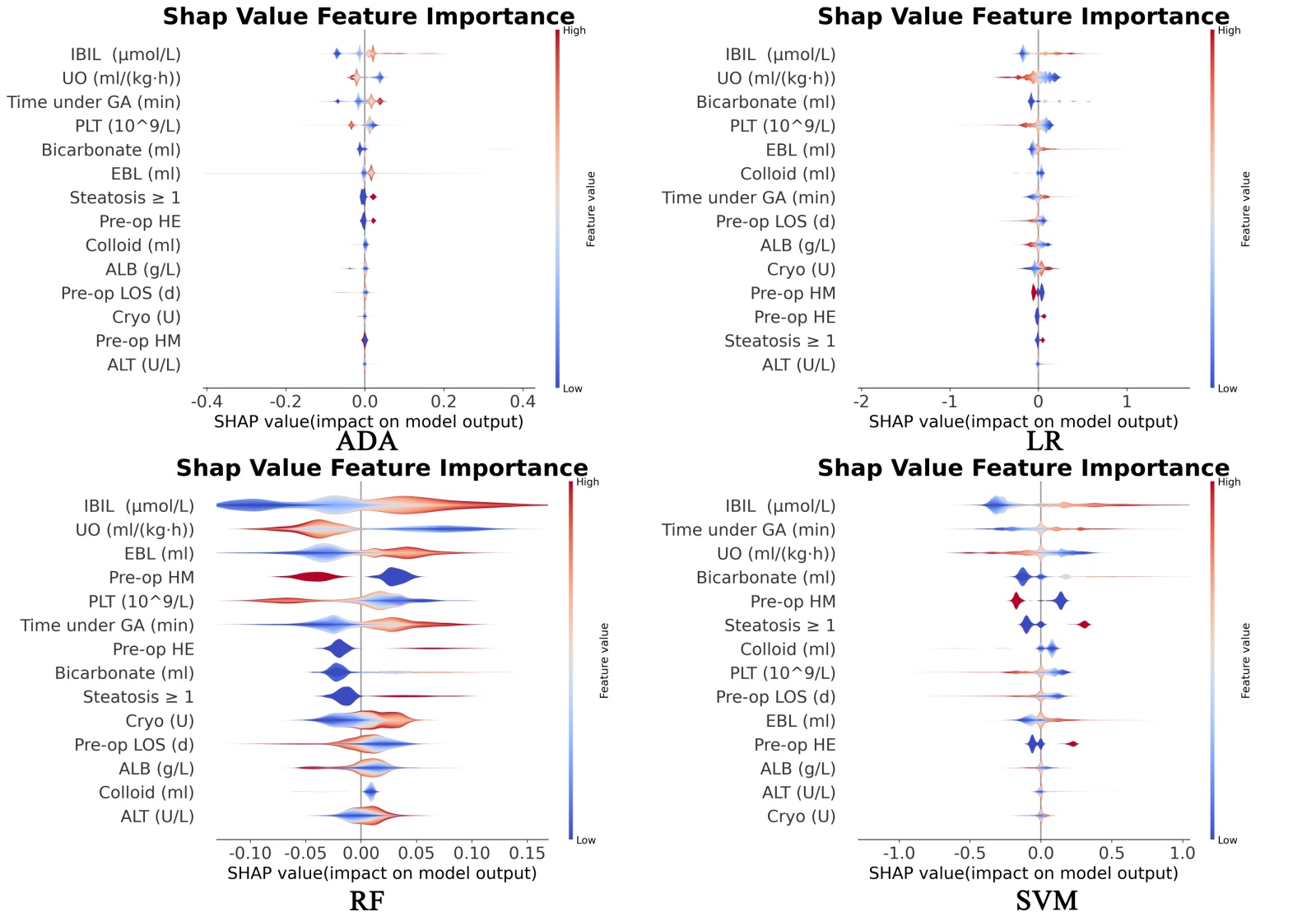


**Figure S2. SHAP summary plot of 4 machine learning models besides GBM. ADA = adaptive boosting; LR = logistic regression; RF = random forest; SVM = support vector machine; GBM = gradient boosting machine.**

1. Kalisvaart M, Schlegel A, Umbro I, de Haan JE, Polak WG, JN IJ, Mirza DF, et al. The AKI Prediction Score: a new prediction model for acute kidney injury after liver transplantation. HPB (Oxford) 2019;21:1707-1717.
