# Supplementary material for: An explainable supervised machine learning predictor of acute kidney injury after adult deceased donor liver transplantation
Source: J Transl Med. 2021 Jul 28;19:321. doi: 10.1186/s12967-021-02990-4 (PMC8317304; doi:10.1186/s12967-021-02990-4)
Supplement: Supplementary file 3 — Additional file 3: Complete Statistics. Table S1. Statistics of the 111 variables that was chosen for initial selection. Table S2. Post-operative medications prior to the diagnosis of AKI or prior to the appearance of maximum SCr in Non-AKI group. Table S3. Stage and time of diagnosis of AKI. Table S4 Coefficient in LASSO analysis of the 38 variables selected by univariate test. [file 12967_2021_2990_MOESM3_ESM.docx]

**Appendix S3 Complete Statistics**

**Table S1 Statistics of the 111 variables that were chosen for initial selection**

|  | **All(N=780)** | **Non-AKI(n=350)** | **AKI(n=430)** | ***P* value** |
| --- | --- | --- | --- | --- |
| **Gender (male, n)** | 682.0(87.436%) | 307.0(87.714%) | 375.0(87.209%) | 0.918 |
| **Age (y)** | 50.719(10.638) | 51.051(10.433) | 50.449(10.808) | 0.295 |
| **Height (cm)** | 167.954(9.065) | 167.734(6.428) | 168.134(10.753) | 0.052 |
| **Weight (kg)** | 64.628(11.304) | 63.404(10.889) | 65.628(11.548) | 0.004 |
| **Body Mass Index** | 22.782(3.574) | 22.539(3.529) | 22.98(3.602) | 0.018 |
| **Preoperative LOS (d)** | 11(2-26) | 14(4-28) | 8(2-23) | 0.001 |
| **Preoperative comorbidities** |  |  |  |  |
| HTN (n) | 82.0(10.513%) | 33.0(9.429%) | 49.0(11.395%) | 0.439 |
| DM (n) | 119.0(15.256%) | 47.0(13.429%) | 72.0(16.744%) | 0.238 |
| MI (n) | 1.0(0.128%) | 0.0(0.0%) | 1.0(0.233%) | 0.918 |
| CAD (n) | 7.0(0.897%) | 2.0(0.571%) | 5.0(1.163%) | 0.625 |
| CKD (n) | 121.0(15.513%) | 49.0(14.0%) | 72.0(16.744%) | 0.34 |
| Smoking (n) | 222.0(28.462%) | 87.0(24.857%) | 135.0(31.395%) | 0.053 |
| Alcohol abuse (n) | 206.0(26.41%) | 85.0(24.286%) | 121.0(28.14%) | 0.257 |
| Previous surgery (n) | 56.0(7.179%) | 25.0(7.143%) | 31.0(7.209%) | 0.917 |
| **Preoperative laboratory values** |  |  |  |  |
| HCT | 0.299(0.076) | 0.312(0.08) | 0.288(0.07) | <0.001 |
| PLT(10^9/L) | 96.026(79.4) | 116.597(95.149) | 79.281(58.79) | <0.001 |
| WBC (10^9/L) | 7.057(5.061) | 6.411(4.143) | 7.583(5.65) | 0.082 |
| ALT (U/L) | 126.282(399.834) | 90.349(235.856) | 155.53(493.081) | 0.004 |
| AST (U/L) | 172.242(538.996) | 148.429(369.227) | 191.626(644.817) | <0.001 |
| TBIL (μmol/L) | 250.278(249.713) | 172.311(217.596) | 313.739(256.351) | <0.001 |
| DBIL (μmol/L) | 159.74(168.516) | 116.107(152.227) | 195.256(172.907) | <0.001 |
| IBIL (μmol/L) | 90.537(96.523) | 56.204(72.764) | 118.483(104.24) | <0.001 |
| ALB (g/L) | 35.668(4.906) | 36.212(5.283) | 35.225(4.535) | 0.023 |
| SCr (μmol/L) | 91.777(70.334) | 92.388(68.852) | 91.28(71.593) | 0.047 |
| BUN (mmol/L) | 6.846(5.823) | 6.56(5.218) | 7.078(6.268) | 0.985 |
| PT (s) | 25.16(13.483) | 21.115(9.851) | 28.452(15.064) | <0.001 |
| APTT (s) | 54.653(20.923) | 49.183(16.041) | 59.105(23.267) | <0.001 |
| FIB (g/L) | 1.982(1.422) | 2.357(1.372) | 1.676(1.39) | <0.001 |
| INR | 2.339(1.574) | 1.912(1.397) | 2.686(1.625) | <0.001 |
| K+ (mmol/L) | 3.851(0.516) | 3.835(0.515) | 3.865(0.517) | 0.504 |
| Na+ (mmol/L) | 138.618(5.11) | 138.737(4.703) | 138.521(5.422) | 0.492 |
| Ca++(mmol/L) | 2.326(0.215) | 2.312(0.193) | 2.337(0.231) | 0.281 |
| HCO3- (mmol/L) | 22.815(4.611) | 22.862(3.354) | 22.777(5.428) | 0.234 |
| eGFR (ml/min*1.73^2) | 95.029(32.145) | 93.749(29.966) | 96.07(33.813) | 0.127 |
| SCr_Mean (μmol/L) | 79.343(71.641) | 75.837(65.256) | 82.197(76.402) | 0.917 |
| **Etiology of liver** |  |  |  |  |
| Hepatitis B (n) | 577.0(73.974%) | 257.0(73.429%) | 320.0(74.419%) | 0.817 |
| Hepatitis C (n) | 17.0(2.179%) | 11.0(3.143%) | 6.0(1.395%) | 0.157 |
| Dual infection (n) | 9.0(1.154%) | 5.0(1.429%) | 4.0(0.93%) | 0.756 |
| Hepatic malignancy (n) | 312.0(40.0%) | 190.0(54.286%) | 122.0(28.372%) | <0.001 |
| Drug-induced liver injury (n) | 7.0(0.897%) | 2.0(0.571%) | 5.0(1.163%) | 0.625 |
| Alcohol-related liver disease (n) | 41.0(5.256%) | 13.0(3.714%) | 28.0(6.512%) | 0.114 |
| Auto-immune hepatitis (n) | 6.0(0.769%) | 1.0(0.286%) | 5.0(1.163%) | 0.326 |
| Hepatolenticular degeneration(n) | 6.0(0.769%) | 2.0(0.571%) | 4.0(0.93%) | 0.874 |
| Hemochromatosis (n) | 0.0(0.0%) | 0.0(0.0%) | 0.0(0.0%) | 1 |
| Cirrhosis (n) | 623.0(79.872%) | 292.0(83.429%) | 331.0(76.977%) | 0.032 |
| Primary biliary cirrhosis (n) | 9.0(1.154%) | 4.0(1.143%) | 5.0(1.163%) | 0.756 |
| Alcoholic liver cirrhosis (n) | 28.0(3.59%) | 9.0(2.571%) | 19.0(4.419%) | 0.236 |
| **Preoperative complications** |  |  |  |  |
| MELD score | 24(22-35) | 22(22-29) | 30(22-38) | <0.001 |
| Portal hypertension (n) | 407.0(52.179%) | 192.0(54.857%) | 215.0(50.0%) | 0.201 |
| Ascites (n) | 321.0(41.154%) | 142.0(40.571%) | 179.0(41.628%) | 0.822 |
| AKI (n) | 172.0(22.051%) | 67.0(19.143%) | 105.0(24.419%) | 0.093 |
| HRS (n) | 33.0(4.231%) | 8.0(2.286%) | 25.0(5.814%) | 0.024 |
| CRRT (n) | 94.0(12.051%) | 24.0(6.857%) | 70.0(16.279%) | <0.001 |
| Frequency of CRRT (times) | 2.567(10.727) | 1.351(8.312) | 3.556(12.269) | <0.001 |
| HE (n) | 180.0(23.077%) | 41.0(11.714%) | 139.0(32.326%) | <0.001 |
| Plasmapheresis (n) | 7.0(0.897%) | 2.0(0.571%) | 5.0(1.163%) | 0.625 |
| HPS (n) | 4.0(0.513%) | 1.0(0.286%) | 3.0(0.698%) | 0.766 |
| ARDS (n) | 7.0(0.897%) | 3.0(0.857%) | 4.0(0.93%) | 0.784 |
| ALI (n) | 0.0(0.0%) | 0.0(0.0%) | 0.0(0.0%) | 1 |
| MV (n) | 49.0(6.282%) | 9.0(2.571%) | 40.0(9.302%) | <0.001 |
| ICU stay (n) | 439.0(56.282%) | 164.0(46.857%) | 275.0(63.953%) | <0.001 |
| Hypokalemia (n) | 183.0(23.462%) | 86.0(24.571%) | 97.0(22.558%) | 0.565 |
| Hyperkalemia (n) | 1.0(0.128%) | 1.0(0.286%) | 0.0(0.0%) | 0.918 |
| Hyponatremia (n) | 163.0(20.897%) | 66.0(18.857%) | 97.0(22.558%) | 0.24 |
| Hypernatremia (n) | 44.0(5.641%) | 10.0(2.857%) | 34.0(7.907%) | 0.004 |
| Hypocalcemia (n) | 0.0(0.0%) | 0.0(0.0%) | 0.0(0.0%) | 1 |
| Hypercalcemia (n) | 31.0(3.974%) | 8.0(2.286%) | 23.0(5.349%) | 0.046 |
| Metabolic acidosis (n) | 336.0(43.077%) | 144.0(41.143%) | 192.0(44.651%) | 0.362 |
| **Donor characteristics** |  |  |  |  |
| Donor age (y) | 39.191(13.966) | 38.894(14.392) | 39.433(13.621) | 0.755 |
| Donor BMI | 22.578(3.199) | 22.336(3.185) | 22.779(3.201) | 0.074 |
| ABO incompatibility (n) | 120.0(15.385%) | 38.0(10.857%) | 82.0(19.07%) | 0.002 |
| Donor Type |  |  |  | 0.248 |
| DBD (n) | 448(57.436%) | 212(60.571%) | 236(54.884%) |  |
| DCD (n) | 324(41.538%) | 134(38.286%) | 190(44.186%) |  |
| DBCD (n) | 8(1.026%) | 4(1.143%) | 4(0.93%) |  |
| Steatosis of donor liver |  |  |  | 0.005 |
| Steatosis grade 0 (n) | 529(67.821%) | 260.0(74.286%) | 269(62.558%) |  |
| Steatosis grade 1 (n) | 170(21.795%) | 62.0(17.714%) | 108(25.116%) |  |
| Steatosis grade 2 (n) | 35(4.487%) | 9.0(2.571%) | 26(6.047%) |  |
| Steatosis grade 3 (n) | 1(0.128%) | 0.0(0.0%) | 1(0.233%) |  |
| Lack of pathology assesment | 45(5.769%) | 19(5.429%) | 26(6.046%) | 0.721 |
| Steatosis grade ≥ 1 (n) | 206.0(26.41%) | 71.0(20.286%) | 135.0(31.395%) | 0.001 |
| Steatosis grade ≥ 2 (n) | 36.0(4.615%) | 9.0(2.571%) | 27.0(6.279%) | 0.022 |
| **Surgery characteristics** |  |  |  |  |
| Time of surgery (min) | 442.713(92.854) | 425.297(87.949) | 456.888(94.418) | <0.001 |
| Time under anesthesia (min) | 538.888(97.864) | 519.56(92.679) | 554.621(99.251) | <0.001 |
| Recipient warm ischemic time (min) | 46.45(12.035) | 45.919(12.183) | 46.883(11.909) | 0.088 |
| Cold ischemic time (h) | 6.255(1.358) | 6.226(1.393) | 6.278(1.329) | 0.476 |
| Complicated hepatic artery reconstruction (n) | 199.0(25.513%) | 81.0(23.143%) | 118.0(27.442%) | 0.206 |
| Choledochojejunostomy (n) | 30.0(3.846%) | 10.0(2.857%) | 20.0(4.651%) | 0.262 |
| Surgical technique |  |  |  | 0.304 |
| Piggyback | 713(91.41%) | 317(90.571%) | 396(92.093%) |  |
| Split liver | 36(4.615%) | 15(4.286%) | 21(4.884%) |  |
| Standard | 31(3.974%) | 18(5.143%) | 13(3.023%) |  |
| **Intraoperative fluid and transfusion** |  |  |  |  |
| Crystalloid (ml) | 2618.423(2240.489) | 2775.575(2366.817) | 2490.944(2126.798) | 0.094 |
| Colloid (ml) | 124.26(427.879) | 153.448(424.742) | 100.583(429.443) | 0.006 |
| Albumin (ml) | 218.295(116.74) | 222.629(111.083) | 214.779(121.15) | 0.483 |
| Other fluids (ml) | 57.356(278.979) | 42.288(240.906) | 69.864(306.747) | 0.097 |
| Transfusion |  |  |  |  |
| RBC (ml) | 1500.39(1318.45) | 1279.989(1333.507) | 1679.177(1280.024) | <0.001 |
| Plasma (ml) | 1862.806(1613.71) | 1725.862(1376.393) | 1973.893(1777.029) | 0.063 |
| Cryoprecipitate (U) | 30.276(15.83) | 27.359(14.9) | 32.653(16.182) | <0.001 |
| EBL (ml) | 2051.489(2027.519) | 1679.857(1890.832) | 2354.685(2086.165) | <0.001 |
| Urine output (ml/(kg*h)) | 3.104(2.146) | 3.708(2.219) | 2.613(1.954) | <0.001 |
| Ascites removal (ml) | 959.665(1889.757) | 947.011(1997.938) | 969.93(1799.531) | 0.196 |
| Gastric drainage (ml) | 56.853(183.966) | 40.402(88.065) | 70.198(233.827) | 0.323 |
| Other estimated fluid loss (ml) | 4.291(50.865) | 3.801(55.227) | 4.69(47.078) | 0.418 |
| **Intraoperative medication** |  |  |  |  |
| rFVIIa (mg) | 0.346(1.127) | 0.211(1.03) | 0.455(1.19) | <0.001 |
| Prothrombin complex concentrate (IU) | 587.692(433.693) | 554.857(434.497) | 614.419(431.7) | 0.043 |
| Fibrinogen (g) | 0.404(1.293) | 0.342(0.735) | 0.453(1.609) | 0.567 |
| Terlipressin (mg) | 0.322(0.551) | 0.195(0.447) | 0.426(0.604) | <0.001 |
| Norepinephrine, bolus (mg) | 0.008(0.022) | 0.006(0.018) | 0.009(0.024) | 0.353 |
| Epinephrine, bolus (mg) | 0.028(0.299) | 0.011(0.161) | 0.042(0.376) | 0.785 |
| Dopamine, bolus (mg) | 12.0(1.538%) | 4.0(1.143%) | 8.0(1.86%) | 0.874 |
| Bicarbonate (ml) | 127.006(234.266) | 89.429(221.225) | 157.593(240.316) | <0.001 |
| Use of norepinephrine, continuous (n) | 649.0(83.205%) | 301.0(86.0%) | 348.0(80.93%) | 0.074 |
| Use of epinephrine, continuous (n) | 553.0(70.897%) | 250.0(71.429%) | 303.0(70.465%) | 0.829 |
| Use of dopamine, continuous (n) | 245.0(31.41%) | 106.0(30.286%) | 139.0(32.326%) | 0.594 |
| Use of aramine (n) | 34.0(4.359%) | 7.0(2.0%) | 27.0(6.279%) | 0.006 |
| **Intraoperative incident** |  |  |  |  |
| Arrhythmia (n) | 761.0(97.564%) | 343.0(98.0%) | 418.0(97.209%) | 0.632 |
| Cardiac arrest (n) | 21.0(2.692%) | 3.0(0.857%) | 18.0(4.186%) | 0.008 |
| Acidosis (n) | 322.0(41.282%) | 133.0(38.0%) | 189.0(43.953%) | 0.108 |
| Hyperlactacidemia (n) | 391.0(50.128%) | 174.0(49.714%) | 217.0(50.465%) | 0.891 |
| Hypokalemia (n) | 301.0(38.59%) | 145.0(41.429%) | 156.0(36.279%) | 0.163 |
| Hypernatronemia (n) | 23.0(2.949%) | 9.0(2.571%) | 14.0(3.256%) | 0.727 |
| Hypotension (n) | 649.0(83.205%) | 298.0(85.143%) | 351.0(81.628%) | 0.226 |

BMI = body mass index; LOS = length of stay; MELD = model for end stage liver disease. CRRT = continuous renal replacement therapy; ARDS = acute respiratory distress syndrome; ICU = intensive care unit; HCT = hematocrit; PLT = platelets; WBC = white blood cell; ALT = alanine transaminase; AST = aspartate transaminase; TBIL = total bilirubin; DBIL = direct bilirubin; IBIL = indirect bilirubin; ALB = albumin; SCr = serum creatinine; BUN = blood urea nitrogen; PT = prothrombin time; APTT = activated partial thromboplastin time; FIB = fibrinogen; INR = international normalized ratio; eGFR = estimated glomerular filtration rate; GA = general anesthesia; RBC = red blood cell; EBL = estimated blood loss; rFVIIa = recombinant activated factor VII.

**Table S2 Post-operative medications prior to the diagnosis of AKI or prior to the appearance of maximum SCr in Non-AKI group**

| **Postoperative medications** | **All(N=780)** | **Non-AKI(n=350)** | **AKI(n=430)** | ***P* value** |
| --- | --- | --- | --- | --- |
| Dose of norepinephrine, post-op (mg/d) | 5.239(12.534) | 4.881(13.185) | 5.53(11.985) | 0.554 |
| Dose of terlipressin, post-op (mg/d) | 0.144(0.425) | 0.098(0.318) | 0.182(0.493) | 0.101 |
| Dose of dopamine, post-op (mg/d) | 44.438(75.749) | 41.684(64.161) | 46.679(84.019) | 0.502 |
| Dose of epinephrine, post-op(mg/d) | 1.848(4.235) | 2.235(5.052) | 1.534(3.403) | 0.001 |
| Succinylated gelatin (n) | 79.0(10.128%) | 43.0(12.286%) | 36.0(8.372%) | 0.092 |
| Hypertonic Sodium Chloride Hydroxyethyl Starch 40 (n) | 5.0(0.641%) | 2.0(0.571%) | 3.0(0.698%) | 0.817 |
| 6% Hydroxyethyl Starch 130/0.4 (n) | 46.0(5.897%) | 25.0(7.143%) | 21.0(4.884%) | 0.238 |
| Use of norepinephrine, post-op, continuous (n) | 142.0(18.205%) | 42.0(12.0%) | 100.0(23.256%) | <0.001 |
| Use of dopamine, post-op, continuous (n) | 177.0(22.692%) | 61.0(17.429%) | 116.0(26.977%) | 0.002 |
| Use of epinephrine, post-op, continuous (n) | 11.0(1.41%) | 4.0(1.143%) | 7.0(1.628%) | 0.79 |
| Vancomycin (n) | 293.0(37.564%) | 143.0(40.857%) | 150.0(34.884%) | 0.101 |
| Imipenem (n) | 141.0(18.077%) | 68.0(19.429%) | 73.0(16.977%) | 0.429 |
| Meropenem (n) | 361.0(46.282%) | 170.0(48.571%) | 191.0(44.419%) | 0.278 |
| Ganciclovir (n) | 317.0(40.641%) | 177.0(50.571%) | 140.0(32.558%) | <0.001 |
| Voriconazole (n) | 22.0(2.821%) | 8.0(2.286%) | 14.0(3.256%) | 0.551 |
| Amphotericin B (n) | 5.0(0.641%) | 1.0(0.286%) | 4.0(0.93%) | 0.502 |
| Caspofungin (n) | 311.0(39.872%) | 158.0(45.143%) | 153.0(35.581%) | 0.008 |
| Micafungin (n) | 59.0(7.564%) | 38.0(10.857%) | 21.0(4.884%) | 0.003 |

AKI = acute kidney injury.

**Table S3 Stage and time of diagnosis of AKI**

|  | **All(N=780)** | **Non-AKI(n=350)** | **AKI(n=430)** | **P_value** |
| --- | --- | --- | --- | --- |
| AKI stage |  |  |  |  |
| No AKI | 350 (44.872%) | 350 (100.0%) |  |  |
| Stage 1 (n) | 177 (22.692%) |  | 177 (41.163%) |  |
| Stage 2 (n) | 63 (8.077%) |  | 63 (14.651%) |  |
| Stage 3 (n) | 190 (24.359%) |  | 190 (44.186%) |  |
| Stage 3 requring CRRT (n) |  |  | 159 (36.977%) |  |
| Post-operative Day |  | Diagnosis of AKI | Appearance of maximum AKI | <0.001 |
| 0 (n) | 574 (73.59%) | 286 (81.714%) | 288 (66.977%) |  |
| 1 (n) | 126 (16.154%) | 39 (11.143%) | 87 (20.233%) |  |
| 2 (n) | 22 (2.821%) | 6 (1.714%) | 16 (3.721%) |  |
| 3 (n) | 16 (2.051%) | 5 (1.429%) | 11 (2.558%) |  |
| 4 (n) | 10 (1.282%) | 0 (0.0%) | 10 (2.326%) |  |
| 5 (n) | 11 (1.41%) | 4 (1.143%) | 7 (1.628%) |  |
| 6 (n) | 12 (1.538%) | 7 (2.0%) | 5 (1.163%) |  |
| 7 (n) | 9 (1.154%) | 3 (0.857%) | 6 (1.395%) |  |

AKI = acute kidney injury; CRRT = continuous renal replacement therapy.

**Table S4 Coefficient in LASSO analysis of the 38 variables selected by univariate test**

| **Variables** | **LASSO coefficient** |
| --- | --- |
| Preoperative LOS (d) | -0.020792273 |
| Time of surgery (min) | 0 |
| Time under GA (min) | 0.034648289 |
| HCT | 0 |
| PLTs (10^9/L) | -0.048625794 |
| ALT (U/L) | 0.005112657 |
| AST (U/L) | 0 |
| IBIL (μmol/L) | 0.081361745 |
| ALB (g/L) | -0.02076109 |
| PT (s) | 0 |
| APTT (s) | 0 |
| FIB (g/L) | 0 |
| INR | 0 |
| Colloid (ml) | -0.038542224 |
| Red blood cell transfusion (ml) | 0 |
| Cryoprecipitate (U) | 0.00096858 |
| EBL (ml) | 0.020612999 |
| rFVIIa (mg) | 0.007784663 |
| Terlipressin (mg) | 0.029397685 |
| Prothrombin complex concentrate (IU) | 0.002731824 |
| Urine output (ml/(kg*h)) | -0.049539459 |
| Bicarbonate (ml) | 0.041176166 |
| Preoperative ICU stay (n) | 0 |
| Mechanical ventilation (n) | 0 |
| Hypernatremia (n) | 0 |
| Hypercalcemia (n) | 0 |
| Cardiac arrest (n) | 0 |
| Hepatic Malignancy (n) | -0.049861925 |
| Cirrhosis (n) | 0 |
| Hepato-renal syndrome (n) | 0 |
| Hepatic encephalopathy (n) | 0.034858742 |
| ABO incompatibility (n) | 0 |
| Use of aramine (n) | 0 |
| Preoperative Frequency of CRRT (times) | 0 |
| Preoperative CRRT (n) | 0 |
| Steatosis grade ≥ 1 | 0.065260215 |
| Steatosis grade ≥ 2 | 0 |
| DBIL (μmol/L) | 0 |
