# Supplementary material for: An explainable supervised machine learning predictor of acute kidney injury after adult deceased donor liver transplantation
Source: J Transl Med. 2021 Jul 28;19:321. doi: 10.1186/s12967-021-02990-4 (PMC8317304; doi:10.1186/s12967-021-02990-4)
Supplement: Supplementary file 4 — Additional file 4: Anesthesia and Immunosuppression Therapy. Appendix S4. Anesthesia and Immunotherapy. [file 12967_2021_2990_MOESM4_ESM.docx]

**Appendix S4: Anesthesia and Immunosuppression Therapy**

The grafts were procured from either donation after circulatory death (DCD), donation after brain death (DBD) or donation after brain death followed by circulatory death (DBCD), with standardized techniques recommended in the guidelines implemented by Chinese Medical Association. **No organs from prosecuted prisoners were used.**

The implantation techniques consisted of piggyback, standard and split liver transplantation techniques. Liver biopsy samples were collected before and after graft reperfusion. Since intraoperative extracorporeal venovenous bypass was not significantly advantageous, preoperative renal replacement therapy was adopted for patients with preoperative renal dysfunction.

Anesthesia was induced by intravenous propofol, sufentanil and cisatracurium, and was maintained with inhalation of sevoflurane or desflurane and continuous infusion of cisatracurium. Arterial-line catheters were inserted into the radial or brachial artery and attached to a Flotrac sensor and monitor (Edwards Lifesciences Corp. US.) to collect invasive arterial blood pressure and cardiac output. Two central venous lines were placed in jugular vein and subclavian vein at either side for central venous pressure monitoring and rapid fluid resuscitation. Transfusion, fluid management and use of vasoactive agents and hemostatic agents were adjusted according to an overall assessment volume balance, hemodynamic stability, estimated blood loss, thromboelastogram blood gas results and cardiac function. Boluses of vasoactive agents were likely to be given to counter immediate circulatory collapse caused by post-reperfusion syndrome, otherwise continuous infusion of vasoconstrictors were preferred. Terlipressin was preferred over norepinephrine when the patient was considered to be at risk of renal dysfunction. Colloids were only considered as a replacement of blood product during reperfusion phase when coagulation deficiency was corrected and satisfactory urine output was observed.

The immunosuppression therapy during surgery included 500 mg of methylprednisolone (1000mg for ABO-incompatible graft) and 20 mg of Basiliximab. The post-operative anti-infectious therapy consisted of broad-spectrum antibiotics, antiviral and antifungal agents. The immunosuppression therapy within 7 days after LT was continued with daily tapered methylprednisolone and a second dose of Basiliximab on Day 4 after the surgery. Tacrolimus introduction was initiated on Day 2 after the surgery for patients receiving ABO-incompatible graft, otherwise it would be initiated at Day 4 to minimize postoperative renal injury. Oral mycophenolate mof (MMF) was usually added after Day 7. Sirolimus would be considered in patients with severe renal dysfunction. However, the decisions for such transition were rare and not consistent among physicians.
