# Supplementary material for: An explainable supervised machine learning predictor of acute kidney injury after adult deceased donor liver transplantation
Source: J Transl Med. 2021 Jul 28;19:321. doi: 10.1186/s12967-021-02990-4 (PMC8317304; doi:10.1186/s12967-021-02990-4)
Supplement: Supplementary file 5 — Additional file 5: Kidney Disease Improving Global Outcomes (KDIGO) diagnostic criteria of AKI. [file 12967_2021_2990_MOESM5_ESM.docx]

**Additional file 6: Kidney Disease Improving Global Outcomes (KDIGO) diagnostic criteria of AKI**

The latest SCr prior to surgery was considered to be a baseline, and SCr between Day 0 to Day 7 post-transplant were collected. AKI was diagnosed when any of the following conditions was met:

1 The highest SCr determined during the first 48 hours after surgery minus the baseline was no less than 26.5 μmol/L or 0.3mg/dL.

2.The highest SCr during the 7 days following the surgery was above 1.5 times the baseline.

3.Patient without prior renal injury or renal disease with identified record of renal replacement therapy after the surgery.

To further elucidate the difference between AKI staging, we also adopted the KDIGO criteria to stage AKI as follows:

Stage 1: Increase in serum creatinine to 1.5 to 1.9 times baseline, or increase in serum creatinine by ≥0.3 mg/dL (≥26.5 μmol/L)..

Stage 2: Increase in serum creatinine to 2.0 to 2.9 times baseline.

Stage 3: Increase in serum creatinine to 3.0 times baseline, or increase in serum creatinine to ≥4.0 mg/dL (≥353.6 μmol/L), or the initiation of renal replacement therapy, or, in patients <18 years, decrease in estimated glomerular filtration rate (eGFR) to <35 mL/min/1.73 m2.
