# Supplementary material for: An explainable supervised machine learning predictor of acute kidney injury after adult deceased donor liver transplantation
Source: J Transl Med. 2021 Jul 28;19:321. doi: 10.1186/s12967-021-02990-4 (PMC8317304; doi:10.1186/s12967-021-02990-4)
Supplement: Supplementary file 6 — Additional file 6: Meld(i) Score. Table S1. Exceptional conditions to be assigned higher MELD score. [file 12967_2021_2990_MOESM6_ESM.docx]

**Additional file 7: MELD(i) score**

Lab MELD(i) score was calculated according to the method proposed by the United Network for Organ Sharing (UNOS) Liver and Intestinal Organ Transplantation Committee (<https://optn.transplant.hrsa.gov/media/1575/policynotice_20151101.pdf>). The detailed formula is as follows.

**Basic Formula**

Candidates who are at least 12 years old receive an initial MELD(i) score equal to:

MELD(i) = 0.957 × ln(Cr) + 0.378 × ln(bilirubin) + 1.120 × ln(INR) + 0.643

Then, round to the tenth decimal place and multiply by 10.

If MELD(i) > 11, perform additional MELD calculation as follows:

MELD = MELD(i) + 1.32 × (137 – Na) – [ 0.033 × MELD(i) × (137 – Na)]

**Additional Rules**

All values in US units (Cr and bilirubin in mg/dL, Na in mEq/L, and INR unitless).

If bilirubin, Cr, or INR is <1.0, use 1.0.

If any of the following is true, use Cr 4.0:

- Cr >4.0.
- ≥2 dialysis treatments within the prior 7 days.
- 24 hours of continuous veno-venous hemodialysis (CVVHD) within the prior 7 days.

If Na <125 mmol/L, use 125. If Na >137 mmol/L, use 137.

Maximum MELD = 40.

**Exceptional Scores**

Some exceptional conditions that could be assigned with additional scores are shown in Table 1.

Table S1 Exceptional conditions to be assigned higher MELD score

| Hepatocellular carcinoma (HCC) | with one lesion between 2 - 5 cm or two to three lesions <3 cm (Milan criteria), provided no vascular invasion or extrahepatic disease. |
| --- | --- |
| Hepatopulmonary syndrome | with PaO2 <60 mmHg on room air. |
| Portopulmonary hypertension | with mean pulmonary artery pressure (mPAP) >25 mmHg at rest but maintained <35 mmHg with treatment |
| Hepatic artery thrombosis | 7 to 14 days post-liver transplantation |
| Familial amyloid polyneuropathy | diagnosed by identification of the transthyretin (TTR) gene mutation by DNA analysis or mass spectrometry in a biopsy sample and confirmation of amyloid deposition in an involved organ. |
| Primary hyperoxaluria | with evidence of alanine glyoxylate aminotransferase deficiency (these patients requires combined liver-kidney transplantation). |
| Cystic fibrosis | with FEV1 (forced expiratory volume in 1 second) <40%. |
| Hilar cholangiocarcinoma. |  |

Table 1 The exceptional conditions that are automatically assigned a MELD Score of 22 (28 in case of hyperoxaluria), with a 10% increase in score every 3 months from diagnosis.
